# O-GlcNAcylation of ZEB1 facilitated mesenchymal pancreatic cancer cell ferroptosis

**DOI:** 10.7150/ijbs.71520

**Published:** 2022-06-21

**Authors:** Xin Wang, Mengqi Liu, Yue Chu, Yanfang Liu, Xiongfeng Cao, Han Zhang, Yao Huang, Aihua Gong, Xiang Liao, Dongqing Wang, Haitao Zhu

**Affiliations:** 1 Laboratory of Medical Imaging, Affiliated Hospital of Jiangsu University, Zhenjiang, China, 212001.; 2 Department of Medical Imaging, Affiliated Hospital of Jiangsu University, Zhenjiang, China, 212001.; 3 School of Medicine, Jiangsu University, Zhenjiang, China, 212013.; 4 Department of Medical Imaging, Affiliated Hospital of Nanjing Medicine University, 211166.; 5 Department of Laboratory Medicine, Affiliated Hospital of Jiangsu University, Zhenjiang, China, 212001.

**Keywords:** Mesenchymal pancreatic cancer cell, ZEB1, O-GlcNAcylation, Ferroptosis, Glycolipid metabolism

## Abstract

**Background:** Mesenchymal cancer cells, resistant to the traditional regulated cell death, are exquisitely vulnerable to ferroptosis. However, the underlying mechanism has been rarely studied. While glycolipid metabolism rewiring is a critical determination of both cancer cell mesenchymal phenotype and cell death resistance, we are interested in the underlying cross talk between glycolipid metabolism and mesenchymal cancer cell ferroptosis sensitivity.

**Methods:** CCK-8, western blot and clone forming assay were used to access the effect of glucose on mesenchymal cancer cell ferroptosis susceptibility and O-GlcNAcylation level. GEPIA database, shRNA knockdown and various pharmacological inhibitors were used to analyze the relationship between O-GlcNAcylation and mesenchymal cancer cell ferroptosis *in vitro* and *in vivo*. A series of experiments were conducted to investigate the underlying mechanisms of glucose induced ZEB1 O-GlcNAcylation on mesenchymal cancer cell ferroptosis susceptibility.

**Results:** Mesenchymal pancreatic cancer cells O-GlcNAcylation level and ferroptosis cell death was significantly increased under high glucose condition *in vitro* and *in vivo*. O-GlcNAcylation of ZEB1, rather than other transcription factors, was involved in this process. Mechanistically, glucose triggered ZEB1 O-GlcNAcylation at Ser555 site enhanced its stabilization and nuclear translocation, induced lipogenesis associated genes, FASN and FADS2, transcription activity, which ultimately resulted in lipid peroxidation dependent mesenchymal pancreatic cancer cell ferroptosis.

**Conclusions:** These results identify a novel role of glycolipid metabolism and O-GlcNAcylation in mesenchymal cancer cells ferroptosis susceptibility, which broaden the molecular mechanism of ferroptosis and suggested a potential clinical therapeutic strategy for refractory tumors.

## Introduction

Mesenchymal state cancer cells exhibited more aggressive behaviors, such as enhanced stemness and metastasis ability, resistance to cell death and traditional therapy avenues 1. Intriguingly, several reports recently revealed that mesenchymal cancer cells were more vulnerable to ferroptosis 2, 3, while the underlying mechanism was still unclear. Thus, deeper investigation of mesenchymal cancer cell ferroptosis represents a potentially clinical therapeutic strategy for refractory tumors.

Distinct from the other forms of regulated cell death (RCD) in its molecular process, ferroptosis is emerging as a novel cell death and driven by iron-dependent phospholipid peroxidation 4. Ferroptosis is regulated by multiple cellular metabolic pathways, including redox homeostasis, mitochondrial activity, and metabolism of amino acids 5. TGF-ZEB1 pathway regulated lipid metabolism has been reported to exhibit increased mesenchymal-type cancer cells susceptibility to ferroptosis 3, 6. Ji-Yoon Lee *et al.* further revealed that polyunsaturated fatty acid biosynthesis pathway also determined the mesenchymal-type gastric cancer cells ferroptosis sensitivity 7. In addition to the lipid and amino acids metabolism, the role of glucose, a major fuel of the energy metabolism, in mesenchymal cancer cell ferroptosis process is currently elusive and deserves deeper investigation.

Glucose and lipid metabolism cross-talk, also termed glycolipid metabolism, is closely related to cancer cell biology, especially in acquiring the mesenchymal property 8. In glioblastoma multiforme, glycolipid metabolism results in the enhancement of malignant mesenchymal phenotype depending on the glycosylation modification 9. Moreover, dysfunction of glycolipid metabolism reprogramming resulted in breast cancer and hepatocellular carcinoma cellular redox homeostasis imbalance, ultimately triggered cell death 10, 11. Due to the central role of redox homeostasis in ferroptotic cell death, we are interested if glycolipid metabolism also involved in mesenchymal cancer cells ferroptosis sensitivity.

In the present study, we confirmed glucose concentrations are positively associated with the mesenchymal pancreatic cancer cell ferroptosis susceptibility and O-GlcNAcylation level *in vitro* and* in vivo*. Furthermore, GlcNAcylation of ZEB1, mesenchymal property transcription factor, at Ser555 site was involved in this process. Finally, we found that inhibited ZEB1 O-GlcNAcylation at Ser555 site attenuated polyunsaturated fatty acid biosynthesis associated genes, FASN and FADS2, transcription activity under high glucose concentration. Thus, our results demonstrated that glucose played a critical role in mesenchymal cancer cells ferroptosis sensitivity, which depended on regulating polyunsaturated fatty acid biosynthesis.

## Methods

### Reagents and plasmids

Erastin (#HY-15763), (1S,3R)-RSL3 (RSL3, #HY-100218A), Ferrostatin-1 (#HY-100579), Z-VAD-FMK (#HY-16658B), Necrosulfonamide (#HY-100573), 6-Diazo-5-oxo-L-nor-Leucine (DON, #HY-108357), UDP-GlcNAc Disodium Salt (UDP-GlcNAc, #HY-112174), Cycloheximide (CHX, #HY-12320), MG-132 (#HY-13259) and Piperazine Erastin (#HY-100887) were purchased from Med Chem Express (MCE, USA). Benzyl 2-acetamido-2-deoxy-a-D-galactopyranoside (BADGP, B4894) was purchased from Sigma-Aldrich (USA). Thiamet-G (TMG, S7213) was purchased from Selleck Chemicals (USA). Human OGT, GFPT1 and ZEB1 knockdown plasmids were cloned into the pPLK GFP lentivirus vector (PPL, China) and FV055 lentivirus vector (Fubio, China), respectively. FV026 lentivirus vector (Fubio, China) was used to overexpress ZEB1-WT and ZEB1 mutant (T678A or S555A). The shRNA and overexpression target sequences are listed in **Table S1 and 2**.

### Cell culture

Human pancreatic cancer cells, PaTU8988, PANC1, ASPC1, BxPC3, mice pancreatic cancer cells, PANC02, human normal pancreatic epithelial cells, HPDE6, human breast cancer cells MCF7, human fibrosarcoma cells HT1080 and human embryonic kidney cells, HEK293T were purchased from the Cell Bank of the China Academy of Sciences (Shanghai, China). PANC1, PaTU8988, ASPC1, HPDE6, HEK293T and MCF7 were cultured in high-glucose Dulbecco's Modified Eagle's medium (DMEM, NanJing KeyGen Biotech, China) supplemented with 10% fetal bovine serum (FBS, ExCell biotech, China) and 1% penicillin-streptomycin (Gibco, USA). BxPC3, PANC02 and HT1080 were propagated in RPMI-1640 Medium with 10% FBS (Gibco, USA) and 1% antibiotics. All cells were cultured at 37 °C in 5% CO_2_ atmosphere. 4.5 kg/L and 1 kg/L was designed for high glucose and glucose depletion condition according to the previously publication 12. All studies were carried out on cells cultured for fewer than 10 passages.

### Cell viability and colony formation assays

Cell viability was measured by Cell Counting Kit-8 kit (CCK-8, Dojindo, Japan). Briefly, cells were cultured into a 96-well plate at a density of 5 × 10^3^ per well for overnight, then treated with indicated agents for the designed time. Subsequently, 90 µl fresh medium and 10 µl CCK-8 solution was added to cells and incubated for 1-4 h at 37 °C. OD value was measured at 450 nm wavelengths by a microplate reader (Bio-tek, USA).

Colony formation assays were performed to evaluate tumorigenic ability. In brief, 1 × 10^3^ cells were seeded into 6-well plates and cultured in complete DMEM medium. The medium was changed every 3 days. After 14 days, the colonies were washed twice with PBS before adding 4% paraformaldehyde (Beyotime, China) and then stained with crystal violet solution (Beyotime, China).

### Establishment of Stable Cell Lines

To establish the cell lines that stably express sh-GFPT1, sh-OGT, sh-ZEB1, ZEB1-WT or ZEB1 mutant (T678A or S555A), PANC1 and PaTU8988 cells were seeded into 24-well plates and infected with lentivirus containing the indicated plasmid followed by 1 µg/ml puromycin for 48 h. All stable cell lines were grown in a DMEM medium containing 10% FBS and 0.75 µg/ml puromycin for further experiments.

### RT-qPCR analysis

Total RNA from cells using FastPure Cell/Tissue Total RNA Isolation Kit (Vazyme Biotech, China) and reverse transcribed to cDNA using HiScript^®^ III RT SuperMix for qPCR (Vazyme Biotech, China), and then mRNA expressions were analyzed by quantitative real-time polymerase chain reaction assay (qRT-PCR) by ChamQ Universal SYBR qPCR Master Mix (Vazyme Biotech, China). The primers were shown in **Table S3**.

### Cell lysis, immunoprecipitation and immunoblotting

Cells were lysed on ice for 30 min using lysis buffer supplemented with PMSF. After centrifugation at 12,000 × g for 10 min at 4 °C, cell lysates were quantified using the bicinchoninic acid (BCA) assay (Vazyme Biotech, China).

For immunoblotting, the 20-30 µg of proteins were separated by SDS polyacrylamide gels (SDS-PAGE) and transferred to PVDF membranes (Bio-Rad, USA). The membrane was blocked with 5% BSA in TBST buffer for 1 h and incubated with the primary antibodies overnight at 4 °C. After 1 h incubation with horseradish peroxidase (HRP)-conjugated secondary antibodies. Membranes were washed three times with TBST buffer. The protein signals were detected by using enhanced chemo luminescence regent (ECL, Millipore, USA). The following primary antibodies were listed in **Table S4**. β-Tubulin were served as the loading control. The software ImageJ (v. 1.8.0, Bethesda, MD, USA) was used for normalized quantification of the western blot 13.

To immunoprecipitate endogenous ZEB1 or WT/mutant ZEB1-Flag for O-GlcNAcylation examination, cells were lysed with Western and IP cell lysis buffer (Leagene biotech, China) supplemented with PMSF. Cell lysates were incubated with indicated primary antibodies overnight at 4 °C. Protein A/G agarose beads (Sigma-Aldrich, USA) were added into cell lysates and incubated at 4 °C for 6 hours. Beads were washed three times with cell lysis buffer. Samples were separated by SDS-PAGE and subjected to immunoblotting with the indicated antibodies.

### Immunofluorescence Staining

Cells were seed into a 24-well plate and fixed with 4% paraformaldehyde for 15 min and permeabilized with 0.3% Triton X-100 for 5 min. The cells were washed with PBS and blocked with 3% BSA in PBST for 1 h at room temperature. After incubation with primary antibodies at 4 °C. The cells were subsequently incubated with Alexa Fluor 594-conjugated secondary antibodies in PBS containing 1% BSA for 1 h. The nucleus was stained with DAPI. After washing with PBST for three times, cells were observed and monitored by fluorescence microscopy (Leica, USA).

### Luciferase Reporter Assay

Human FADS2 promoter (from -2000 to 0 bp) was subcloned into the pGL3 vector (Promega). HEK293T cells were seeded into a 6-well plate and transfected with ZEB1-overexpression, FADS2 promoter and renilla plasmids the next day. After 36 hours, luciferase activity was performed using the Dual-Luciferase Reporter Assay Kit (Vazyme, China) according to manufacturer's instruction. Firefly luciferase activity was normalized to renilla luciferase activity.

### CHX assay for protein stability

For CHX assay, PANC1 and PaTU8988 cells were seeded into 6-well plates. Next day, Cycloheximide (CHX) was added at a dose of 100 µg/ml. Cells were lysed after the treatment of 0, 3, 6, 12 and 18 h, respectively. 20-30 µg protein was used to perform by western blot.

### Immunohistochemistry (IHC)

The tissue microarrays were obtained from Servicebio (Wuhan, China). The tissue microarrays were deparaffinized by treating with xylene (15 min, three times) and then hydrated by performing 100%, 90%, 70% ethanol for 5 min respectively. The slides were performed antigen retrieval by steaming with PH 6.0 sodium citrate solution and blocked in Dako serum blocker for 45 min. Slide were incubated with anti-O-Linked N-Acetylglucosamine antibody (1:100, Abcam) overnight at 4 °C. Slides were stained with secondary antibody for 30 min followed by incubation with HRP-conjugated streptomyces ovalbumin. DAB detection kit was utilized to monitor primary antibody levels.

### Xenograft tumor models

Female C57BL/6J mice (4-6 weeks) were obtained from the Changzhou Cavens Laboratory Animal Company. All the animal experiments were performed under the standard conditions in Animal Center of Jiangsu University and by the Committee on the Use of Live Animals for Teaching and Research of the Jiangsu University. DMSO or Thiamet-G (20 µM) pre-treated PANC02 cells were injected subcutaneously into the right posterior flank of each C57BL/6J mice, respectively. Tumor-bearing mice were injections with DMSO or piperazine Erastin (40 mg/kg) every other day for 2 weeks. Tumor's volumes were calculated with the formula length × width^2^ × 1/2. Tumor growth inhibition percent = (the mean tumor size of control group - the mean tumor size of treatment group) / the mean tumor size of control group × 100 %.

### Lipid peroxidation assay

Lipid peroxidation refers to the oxidative degradation of lipids which produces free radicals in cell membranes and contributes to ferroptosis. Malondialdehyde (MDA) and 4-hydroxynonenal (4-HNE), the end production of lipid peroxidation, was used as markers of lipid peroxidation. The relative MDA concentration from tumor or cell lysates were used to measure by Lipid Peroxidation Assay Kit (Abcam, ab118970, USA). Briefly, the MDA in the lysates reacted with thiobarbituric acid (TBA) to generate an MDA-TBA adduct which was quantified calorimetrically. OD value was measured at 532 nm wavelengths by using a microplate reader (Bio-tek, USA).

### Statistical Analysis

All results are expressed as the mean ± SEM. The significance between groups was analyzed by Student's *t* test and one-way analysis of variance (ANOVA) and values of *p* < 0.05 were considered to reflect statistically significant difference. All samples were independent biological replicates.

## Results

### Glucose enhanced mesenchymal pancreatic cancer cells ferroptosis sensitivity and O-GlcNAcylation level

**Figure 1:**
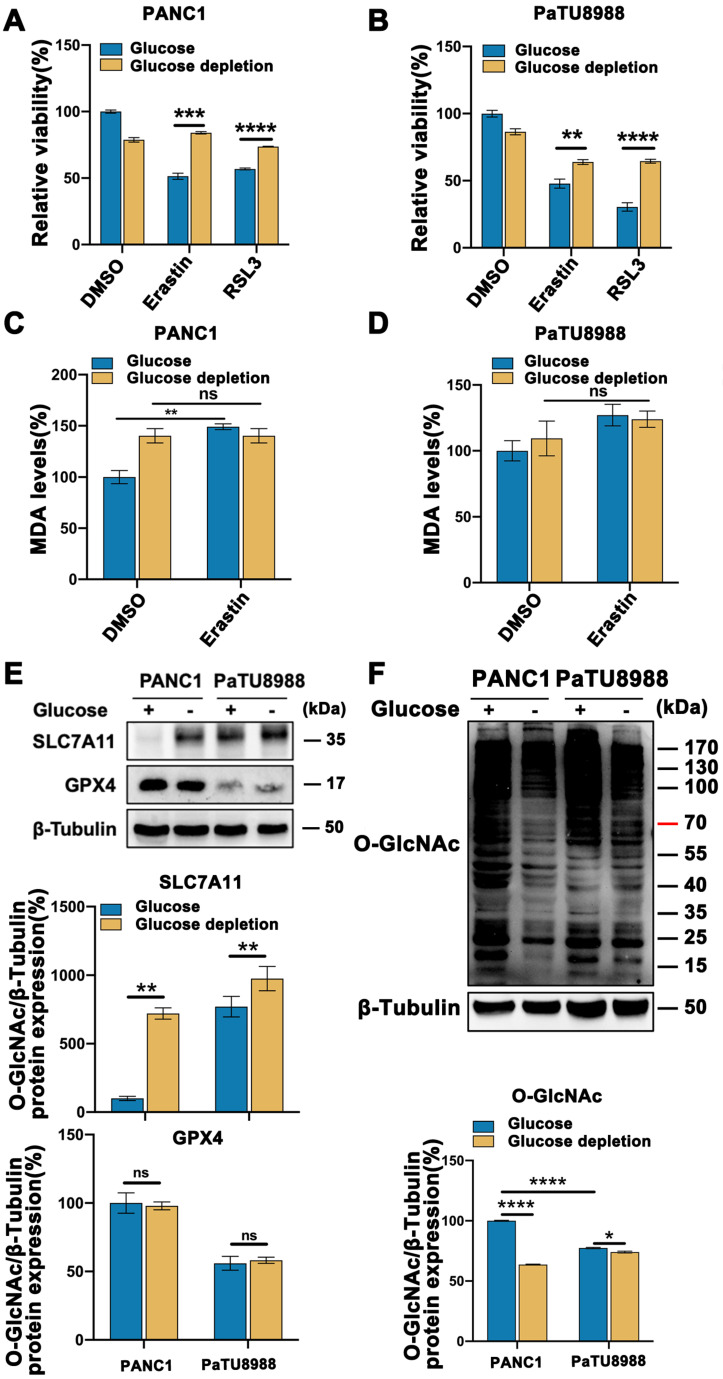
**Glucose enhanced mesenchymal pancreatic cancer cells ferroptosis sensitivity and O-GlcNAcylation level.** (**A-B**) CCK-8 assay assessed the cell viability of PANC1 (**A**) and PaTU8988 (**B**) cells treated with Erastin (4, 15 µM) or RSL3 (0.5 µM) for 48 h in glucose and glucose depletion medium, respectively. (**C-D**) MDA level in PANC1 (**C**) and PaTU8988 (**D**) cells treated with Erastin (4, 15 µM) in glucose and glucose depletion medium for 48 h. (**E**) Western blot analyzed the SLC7A11 expression at the protein level in PANC1 and PaTU8988 cells cultured with glucose or glucose-depletion medium for 48 h. β-Tubulin was used as a loading control. The ratios between SLC7A11/GPX4 and β-Tubulin protein levels were shown, and the PANC1 cells underlying glucose present medium were set to 100%. (**F**) Western blot showed the O-GlcNAcylation level in PANC1 and PaTU8988 cells cultured with glucose or glucose-depletion medium for 48 h. β-Tubulin was used as a loading control. The ratios between O-GlcNAc and β-Tubulin protein levels were shown, and the PANC1 cells underlying glucose present medium were set to 100%. Experiments were repeated three times and the data are expressed as the mean ± SEM. ∗*P* < 0.05. ∗∗*P* < 0.01. ∗∗∗*P* < 0.001. ∗∗∗∗*P* < 0.0001.

To assess the mesenchymal property of pancreatic cancer cell lines, we evaluated the expression level of N-cadherin, a mesenchymal marker, and E-cadherin, an epithelial marker, according to the previously publication 14. Among the pancreatic cell line panel, PANC1, PaTU8988 and PANC02 expressed high expression level of N-cadherin and low level of E-cadherin (**Figure S1A**), which were used in the following experiments. To determine the role of glucose in mesenchymal cancer cell ferroptosis, high glucose and glucose depleted medium cultured PANC1 and PaTU8988 were treated with Erastin and RSL3, two classical ferroptosis inducers, respectively. CCK-8 assay revealed that Erastin and RSL3 could significantly induce the cancer cell death, which could be restored by ferroptosis inhibitor, ferrostatin-1, but not by apoptotic inhibitor ZAVD-FMK or necroptosis inhibitor Necrosulfonamide, indicating the specificity of the two ferroptosis inducers (**Figure S1B**). Compared to the low glucose culture condition, high glucose enhanced Erastin and RSL3 induced PANC1 and PaTU8988 cells ferroptosis (**Figure 1A-B**). Also. the level of malondialdehyde (MDA), an end product of lipid peroxidation and positively ferroptosis level indicator, was significantly increased in high glucose cultured PANC1 and PaTU8988 cells following Erastin treatment (**Figure 1C-D, Figure S1C**). SLC7A11, a negatively ferroptosis indicator and involved in the crosstalk between cystine and glutamate, expression level down-regulated in the high glucose cultured PANC1 and PaTU8988 cells (**Figure 1E, Figure S1D**). GPX4 expression level unchanged under high glucose or glucose-depleted condition (**Figure 1E**). These results suggested that glucose concentration was positively correlation to the ferroptosis sensitivity of mesenchymal pancreatic cancer cells.

These observations prompted us to identify the new connection between glucose and mesenchymal pancreatic cancer cells ferroptosis sensitivity. As O-GlcNAcylation is a special glucose sensor, we next wonder whether O-GlcNAcylation is involved in this process. Accordingly, O-GlcNAcylation level significantly increased in the high glucose cultured PANC1 and PaTU8988 cells (**Figure 1F, Figure S1E-F**). Moreover, according to the previously publications, the mesenchymal human fibrosarcoma HT1080 cells 15, epithelial human breast cancer MCF7 cells 16 and human normal pancreatic epithelial cell line HPDE6 were chosen for the repeat experiments. The similar results can be acquired from mesenchymal HT1080 cells (**Figure S1I-J**), rather than epithelial MCF7 cells (**Figure S1I-J**) and normal HPDE6 cells (**Figure S1G-H**). Thus, these findings demonstrate that O-GlcNAcylation may involve in ferroptosis in mesenchymal cancer cells.

### Glucose induced O-GlcNAcylation promoted mesenchymal pancreatic cancer cells ferroptosis

**Figure 2:**
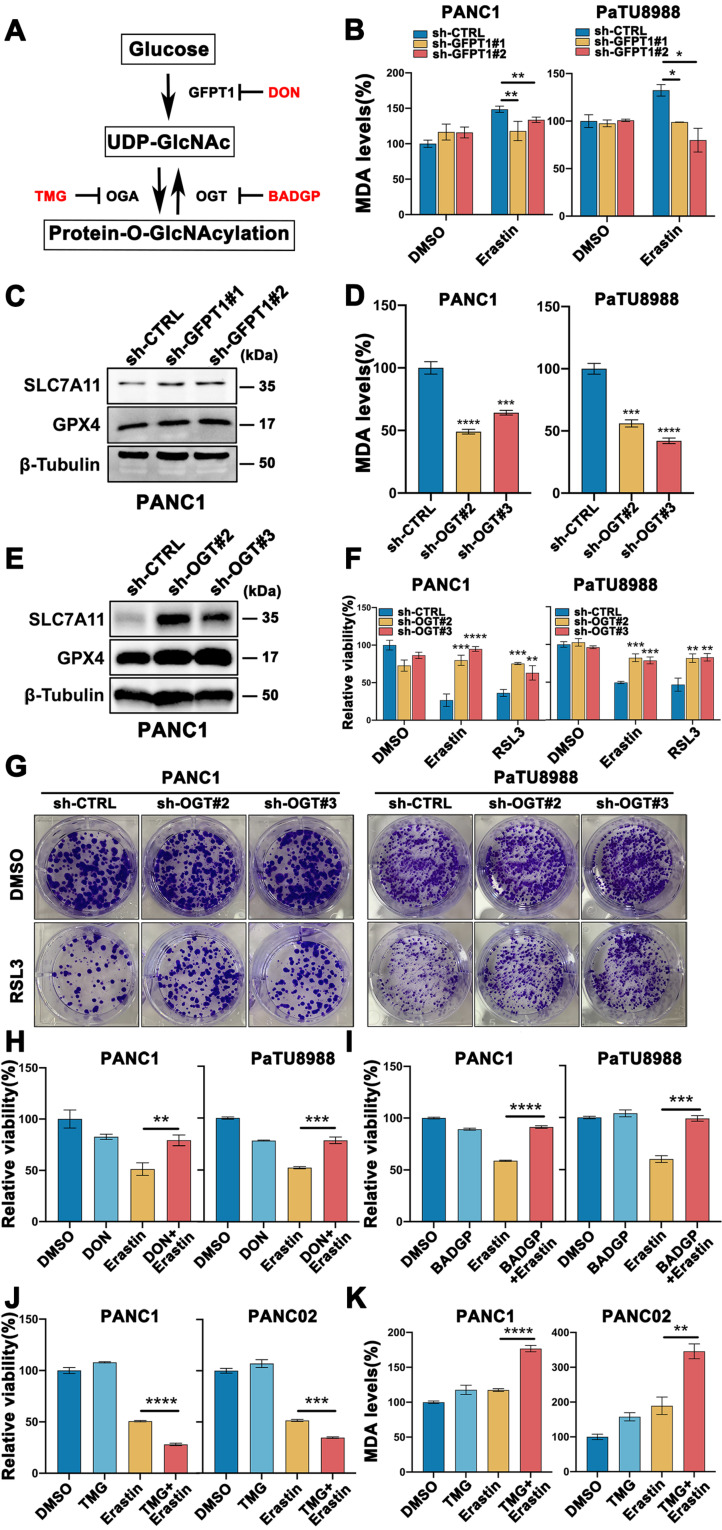
** Glucose induced O-GlcNAcylation promoted mesenchymal pancreatic cancer cells ferroptosis.** (**A**) Schematic of the O-GlcNAcylation metabolism in cancer cells. (**B**) MDA level in GFPT1-silenced (sh-GFPT1) or control (sh-CTRL) PANC1 and PaTU8988 cells treated with Erastin (4, 15 µM) for 48 h. (**C**) Western Blot assessed the SLC7A11 and GPX4 levels in GFPT1-silenced or control (sh-CTRL) PANC1 cells. β-Tubulin was used as a loading control. (**D**) MDA level in OGT-silenced (sh-OGT) or control (sh-CTRL) PANC1 and PaTU8988 cells. (**E**) Western Blot assessed the SLC7A11 and GPX4 levels in OGT-silenced (sh-OGT) or control (sh-CTRL) PANC1 cells. β-Tubulin was used as a loading control. (**F**) CCK-8 assessed the cell viability of OGT-silenced (sh-OGT) or control (sh-CTRL) PANC1 and PaTU8988 cells treated with Erastin (4, 15 µM) or RSL3 (0.5, 5 µM) for 48 h. (**G**) Colony forming ability of OGT-silenced (sh-OGT) or control (sh-CTRL) PANC1 and PaTU8988 cells treated with RSL3 (0.5, 5 µM). (**H**) Cell viability of PANC1 and PaTU8988 cells treated with DMSO, Erastin (4, 15 µM) combined with DON (10 µM). (**I**) Cell viability of PANC1 and PaTU8988 cells treated with DMSO, Erastin (4, 15 µM) in the presence or absence of BADGP (5 mM). (**J**) Cell viability of PANC1 and PANC02 treated with DMSO, Erastin (4, 15µM) in the presence or absence of Thiamet-G (20 µM). (**K**) MDA level of PANC1 and PANC02 cells with the treatments DMSO, Erastin (4, 15µM) in the presence or absence of Thiamet-G (20 µM). TMG represents for Thiamet-G. DON represents for 6-Diazo-5-oxo-L-nor-Leucine. BADGP represents for Benzyl 2-acetamido-2-deoxy-a-D-galactopyranoside. RSL3 represents for (1S,3R)-RSL3. MDA represents for malondialdehyde. O-GlcNAc represents for O-GlcNAcylation. Experiments were repeated three times and the data are expressed as the mean ± SEM. ∗*P* < 0.05. ∗∗*P* < 0.01. ∗∗∗*P* < 0.001. ∗∗∗∗*P* < 0.0001.

Cellular O-GlcNAcylation is regulated by O-GlcNAc-cycling enzymes, GFPT1, the key enzyme catalyzes the production of UDP-GlcNAc by regulating hexosamine biosynthesis pathway (HBP), OGT and OGA, a pair of interactional enzymes catalyze the addition and removal of UDP-GlcNAc, as show in **Figure 2A**. To further elucidate the functional role of O-GlcNAcylation in mesenchymal pancreatic cancer cells ferroptosis, stable GFPT1 (GFPT1 shRNA1 and shRNA2) and OGT (OGT shRNA2 and shRNA3) knockdown cell clones were established with high silencing efficiency confirmed by western blotting (**Figure S2A-B**). Also, the O-GlcNAcylation expression level was significantly decreased in GFPT1- and OGT-silenced cells compared to parental cells (**Figure S2C-D**). SLC7A11 and GPX4, the direct target of class II ferroptosis-inducing components (FINs) and represent a hurdle limiting potency of ferroptotic cell death, protein expression level increased (**Figure 2C, E and Figure S2E-H**), and MDA level was significantly reversed (**Figure 2B and D**) in GFPT1- and OGT-silenced cells. Also, Erastin and RSL3 induced ferroptosis (**Figure 2F**) and colony forming capability (**Figure 2G**) was significantly reversed in OGT-silenced cells.

To further confirm these findings, PANC1 and PaTU8988 cells were treated with GFPT1 and OGT inhibitors, DON and BADGP, respectively, and similar results can be acquired (**Figure 2H-I and Figure S2I**). Moreover, Thiamet-G, the inhibitor of O-GlcNAcase (OGA) which catalyzed the reverse function of OGT, to enhance O-GlcNAcylation, was also used (**Figure S2J**). Combined Thiamet-G and Erastin treatment significantly induced cell death (**Figure 2J**) and enhanced MDA level (**Figure 2K**) in glucose-depleted condition. These findings suggest that O-GlcNAcylation was involved in glucose induced mesenchymal pancreatic cancer cells ferroptosis sensitivity.

### O-GlcNAc modification of ZEB1 is involved in glucose regulated mesenchymal pancreatic cancer cells ferroptosis sensitivity

**Figure 3:**
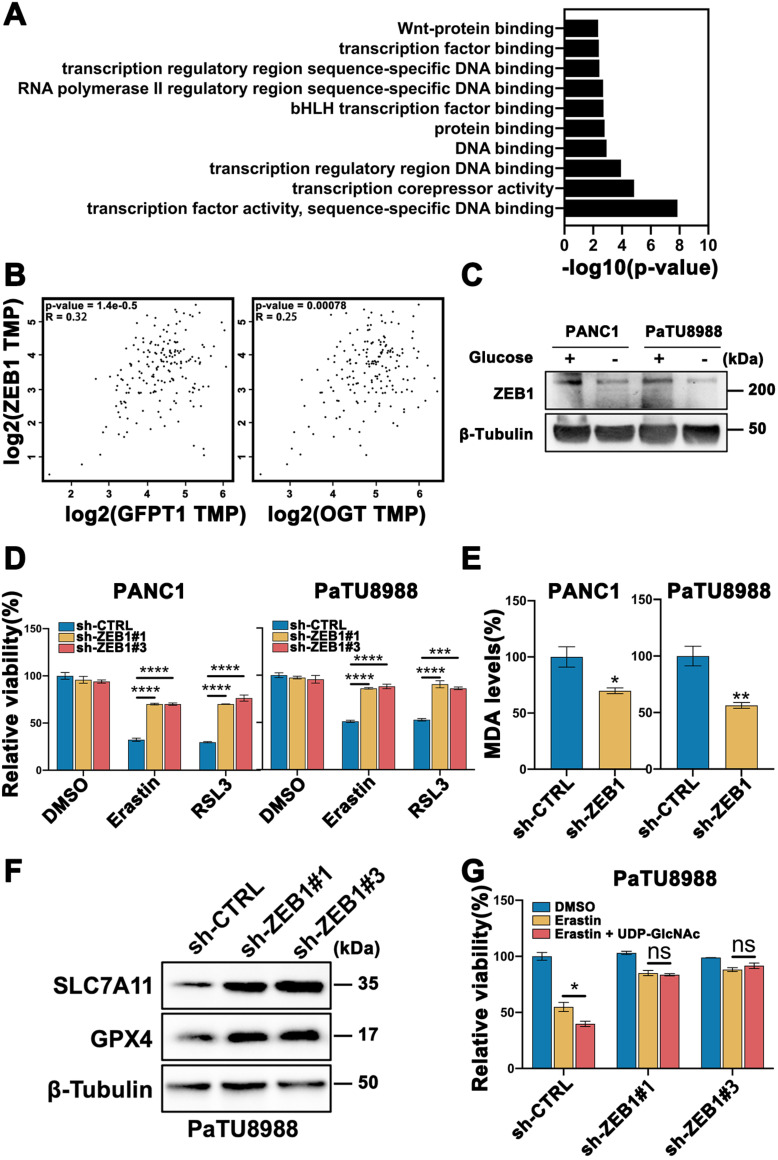
**O-GlcNAc modification of ZEB1 is involved in glucose regulated mesenchymal pancreatic cancer cells ferroptosis sensitivity.** (**A**) List of canonical pathways alteration in OGT-inhibited pancreatic cancer cells. (**B**) GEPIA analyzed the correlation between ZEB1 and O-GlcNAcylation-associated genes expression from the Cancer Genome Atlas (TCGA) PDAC samples. (**C**) Western blot assessed the ZEB1 expression level in PANC1 and PaTU8988 cells cultured in glucose or glucose-depletion medium. β-Tubulin was used as a loading control. (**D**) Cell viability of ZEB1 knockdown PANC1 and PaTU8988 cells treated with Erastin (4, 15 µM) or RSL3 (0.5, 5 µM). (**E**) MDA level in sh-CTRL or sh-ZEB1 PANC1 and PaTU8988 cells. (**F**) Western blot assessed SLC7A11 and GPX4 levels in ZEB1-silenced (sh-ZEB1) or control (sh-CTRL) PANC1 cells. β-Tubulin was used as a loading control. (**G**) Cell viability of ZEB1 knockdown PaTU8988 cells treated with Erastin (15 µM) with or without UDP-GlcNAc treatment. RSL3 represents for (1S,3R)-RSL3. MDA represents for malondialdehyde. UDP-GlcNAc represents for UDP-GlcNAc Disodium Salt. Experiments were repeated three times and the data are expressed as the mean ± SEM. ∗*P* < 0.05. ∗∗*P* < 0.01. ∗∗∗*P* < 0.001. ∗∗∗∗*P* < 0.0001.

To further explore the mechanism of O-GlcNAcylation promoted ferroptosis, we analyzed a gene expression database using the OGT knockout pancreatic cancer cells in Gene Expression Omnibus (GEO: accession numbers GSE114472) 17. The Kyoto Encyclopedia of Genes and Genomes (KEGG) analysis found that the majority of the differential genes belonged to transcription factor activity set in OGT-knockout cells compared with control cells (**Figure 3A**). Mesenchymal property of cancer cell is closely regulated by epithelial-mesenchymal transition (EMT) related transcription factors. To ascertain which transcription factor indeed plays a key role in the mesenchymal pancreatic cancer cells ferroptosis, we firstly analyzed the patients' data in GEPIA database (https://gepia.cancer-pku.cn/index.html) and identified that ZEB1 (**Figure 3B**), rather than Twist, Snail, and Snai2 (**Figure S3A**), are highly correlated with O-GlcNAcylation critically associated genes (GFPT1 and OGT). Thus, we speculate that ZEB1 O-GlcNAcylation may be involved in glucose regulated mesenchymal pancreatic cancer cells ferroptosis sensitivity.

PANC1 and PaTU8988 also expressed higher level of ZEB1 (**Figure S1A**) among the pancreatic cancer cell line panel, which was decreased in glucose depletion culture condition (**Figure 3C, Figure S1D**). Secondly, we constructed ZEB1 silenced cell lines (**Figure S3B**) and cultured under high glucose condition. Knockdown of ZEB1 significantly increased cell viability in the presence of Erastin and RSL3 (**Figure 3D**). Moreover, decreased MDA level (**Figure 3E**) and increased SLC7A11 and GPX4 expression levels (**Figure 3F and Figure S3C**) can be observed in ZEB1 silenced PANC1 and PaTU8988 cells. Next, to exam the role of ZEB1 in glucose regulating ferroptosis, the lipid peroxidation in ZEB1 knockdown PaTU8988 cells were measured. MDA assay showed that ZEB1 knockdown exhibited decreased lipid peroxidation level cultured with glucose depleted medium compared with glucose medium (**Figure S3D**). Moreover, O-GlcNAcylation enhanced mesenchymal pancreatic cancer cells ferroptosis was diminished in ZEB1 knockdown cells (**Figure 3G**). These results indicated that ZEB1 is a critical regulator of O-GlcNAcylation on mesenchymal pancreatic cancer cell ferroptosis.

**Figure 4:**
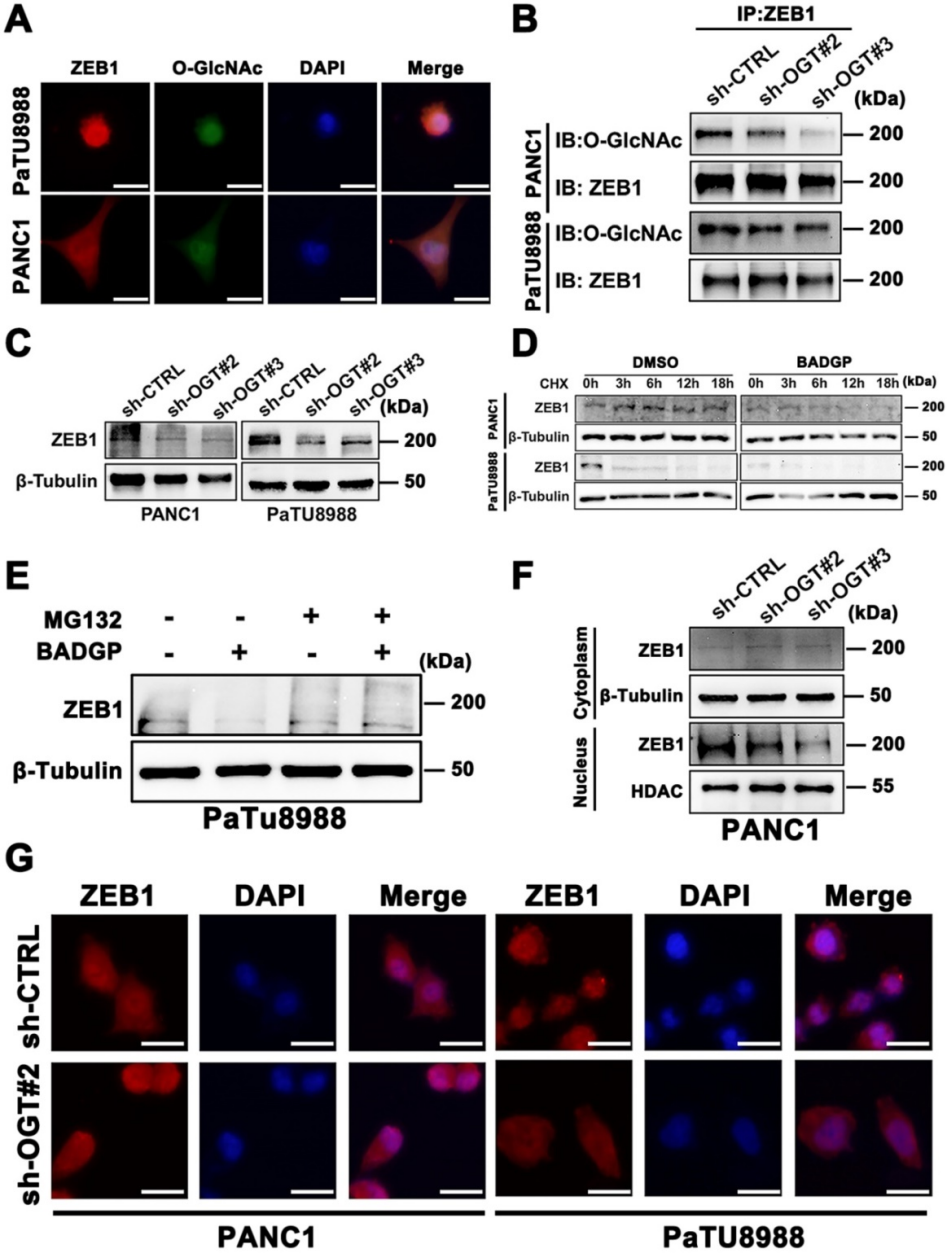
** O-GlcNAc modification determines ZEB1 stabilization and nuclear translocation.** (**A**) Immunofluorescence images of PANC1 and PaTU8988 cells stained with anti-ZEB1, anti-O-GlcNAc antibodies and DAPI solution. Scale bar, 25 µm. (**B**) ZEB1 O-GlcNAcylation was determined by Co-IP in OGT-silenced or control (sh-CTRL) PANC1 and PaTU8988 cells. (**C**) PANC1 and PaTU8988 cells were transfected with sh-CTRL or sh-OGT plasmid and Western blot assessed the expression level of ZEB1 in OGT-silenced PANC1 and PaTU8988 cells. β-Tubulin was used as a loading control. (**D**) PANC1 and PaTU8988 were performed CHX chase analysis for indicated time points (0, 3, 6, 12, 18 h) with or without BADGP (5 mM). (**E**) Western blot assessed the ZEB1 expression level in PaTU8988 cells treated MG-132 with or without BADGP (5 mM). β-Tubulin was used as a loading control. (**F**) Western blot assessed the expression of ZEB1 in PNAC1 cells cytoplasm and nucleus fractions. β-Tubulin was used as a cytoplasm loading control, and HDAC was used as a nucleolar loading control. (**G**) Immunofluorescence images of PANC1 and PaTU8988 OGT knockdown cells stained with anti-ZEB1 antibodies and DAPI solutions. Scale bar, 25 µm. O-GlcNAc represents for O-GlcNAcylation. DAPI represents for fluorescence staining image of nucleus. Merge represents for combined fluorescence staining image of ZEB1, O-GlcNAc and DAPI. CHX represents for Cycloheximide. BADGP represents for Benzyl 2-acetamido-2-deoxy-a-D-galactopyranoside. Experiments were repeated three times and the data are expressed as the mean ± SEM.∗ *P* < 0.05. ∗∗ *P* < 0.01. ∗∗∗ *P* < 0.001. ∗∗∗∗ *P* < 0.0001.

We next wonder the exact role of O-GlcNAcylation in ZEB1 expression and function. Immunofluorescence assays confirmed the co-localization of ZEB1 and O-GlcNAcylation (**Figure 4A**). Immunoprecipitation assays further confirmed the existing of O-GlcNAcylation on ZEB1 (**Figure S3E**) and knockdown OGT can effectively decrease the extent of O-GlcNAcylation on ZEB1 (**Figure 4B**). ZEB1 expression at the protein level (**Figure 4C**), rather than mRNA level (**Figure S3F**), decreased in OGT-silenced cells. Moreover, Thiamet-G treatment increased the ZEB1 protein levels, while BADGP decreased ZEB1 level (**Figure S3G**). These results indicated that O-GlcNAcylation regulate ZEB1 expression depending on the translation or post-translation manner. We next explored whether O-GlcNAcylation was involved in the modulation of ZEB1 protein stability. Cycloheximide (CHX) chase analyzed revealed that the protein stability of ZEB1 was impaired in the presence of BADGP (**Figure 4D**), and this phenomenon was blocked by MG132 (**Figure 4E**), a cell-permeable inhibitor which suppresses the degradation of ubiquitinated proteins in proteasome.

To extend these observations, we examined localization of ZEB1 in control and O-GlcNAcylation inhibited pancreatic cancer cells. Compared to the control cells, the nuclear localization of ZEB1 was decreased in O-GlcNAcylation inhibition pancreatic cancer cells as shown by Immunoblotting and Immunofluorescence assays (**Figure 4F-G**). These results suggested that O-GlcNAcylation could stabilize the ZEB1 at translation or post-translation level and further promote its nuclear translocation.

### ZEB1 O-GlcNAcylation at Ser555 contributes to glucose driven mesenchymal pancreatic cancer cells ferroptotic cell death

**Figure 5:**
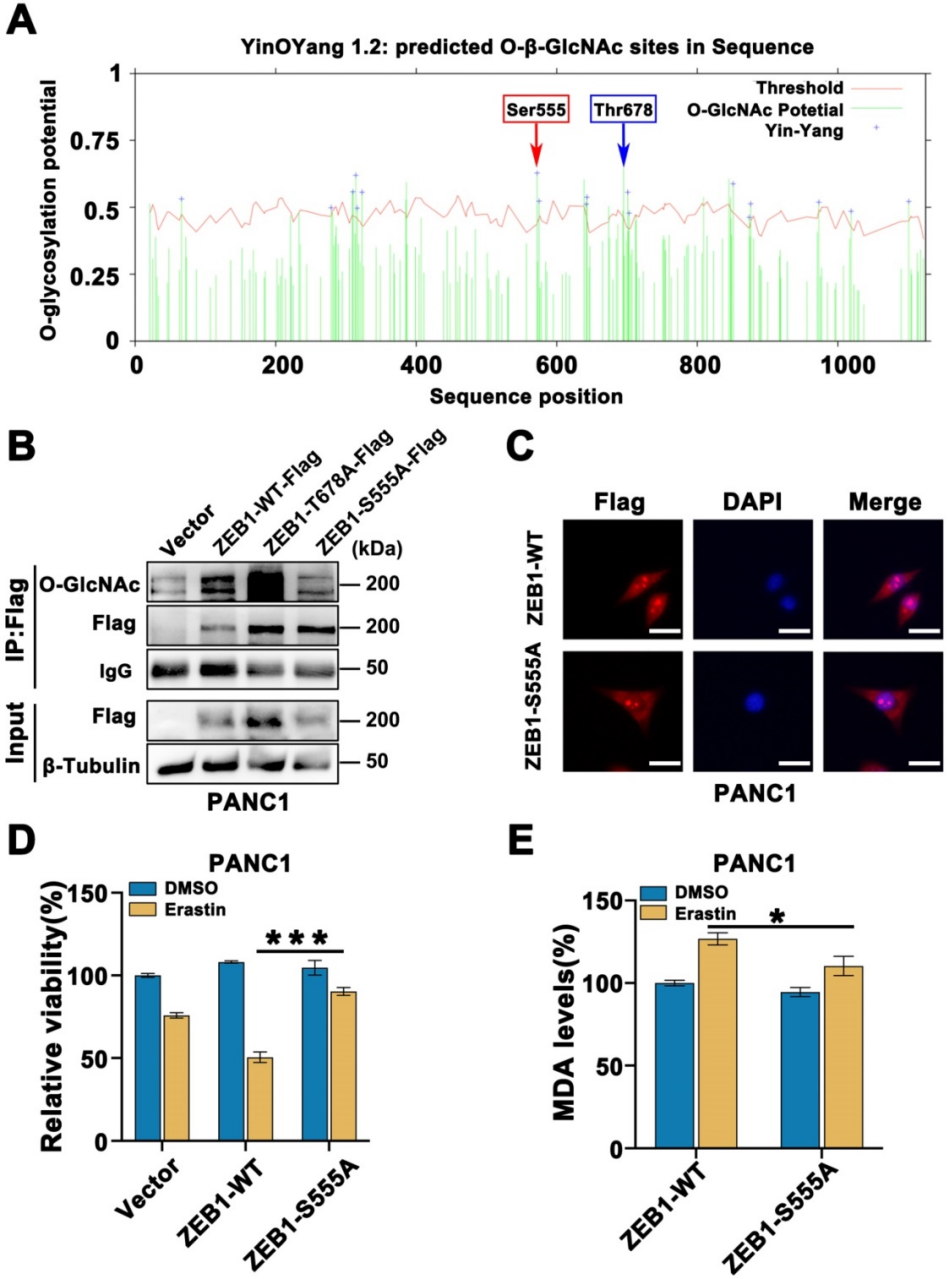
**ZEB1 O-GlcNAcylation at Ser555 contributes to glucose driven mesenchymal pancreatic cancer cells ferroptotic cell death.** (**A**) YinOYang 1.2 server predicts O-GlcNAcylation site of ZEB1. (**B**) Co-IP analysis of ZEB1 O-GlcNAcylation in PANC1 cells transfected with Vector, ZEB1-WT-Flag, ZEB1-T678A-Flag and ZEB1-S555A-Flag plasmids. β-Tubulin was used as a loading control. (**C**) Immunofluorescence images of PANC1 cells transfected with ZEB1-WT-Flag and ZEB1-S555A-Flag plasmids and stained with Flag antibodies and DAPI solutions. (**D**) PANC1 cells were transfected with indicated plasmids, and cell viability was detected by CCK-8 assay under the treatment Erastin (4 µM) for 48 h. (**E**) PANC1 cells were transfected with indicated plasmids, followed by Erastin (4 µM) treatment for 48 h. Relative lipid peroxidation levels was measured through MDA assay kit. O-GlcNAc represents for O-GlcNAcylation. DAPI represents for fluorescence staining image of nucleus. Merge represents for combined fluorescence staining image of Flag and DAPI. MDA represents for malondialdehyde. Experiments were repeated three times and the data are expressed as the mean ± SEM. ∗* P* < 0.05. ∗∗ *P* < 0.01. ∗∗∗ *P* < 0.001. ∗∗∗∗ *P* < 0.0001.

To understand the deeper mechanisms responsible for the ZEB1 O-GlcNAcylation regulation ferroptosis, we searched the potential O-GlcNAcylation sites in ZEB1 that involved in this process. Using the YinOYang 1.2 server (http://www.cbs.dtu.dk/services/YinOYang/), we identified Thr678 and Ser555 site with the highest score that could be directly modified by O-GlcNAcylation (**Figure 5A**). To determine the importance of two O-GlcNAcylation sites on ZEB1, we generated T678A and S555A mutants by mutating the amino acid at the sites of Flag-ZEB1 plasmid to alanine. Co-IP assay showed that O-GlcNAcylation expression level was markedly reduced in the S555A mutant, rather than ZEB1 wide type and T678A mutant (**Figure 5B and Figure S4A**). Moreover, immunofluorescence assays revealed that ZEB1 S555A mutant decreased nuclear translocation of ZEB1 (**Figure 5C**). These results suggested that S555 site on ZEB1 is critical important for O-GlcNAcylation and nuclear localization in the glucose cultured PANC1 and PaTU8988 cells.

Furthermore, compared to the ZEB1 wide type cells, ZEB1-S555A mutant cancer cells displayed the increased cell viability (**Figure 5D**) and decreased the levels of MDA (**Figure 5E and Figure S4B**) in the presence of Erastin under the glucose cultured condition. These results indicated that ZEB1 O-GlcNAcylation at S555 site determined the sensitivity of mesenchymal cancer cell ferroptosis under the glucose condition.

### FASN-FADS2 axis regulated polyunsaturated fatty acid biosynthesis was involved in ZEB1 O-GlcNAcylation driven ferroptosis sensitivity

**Figure 6:**
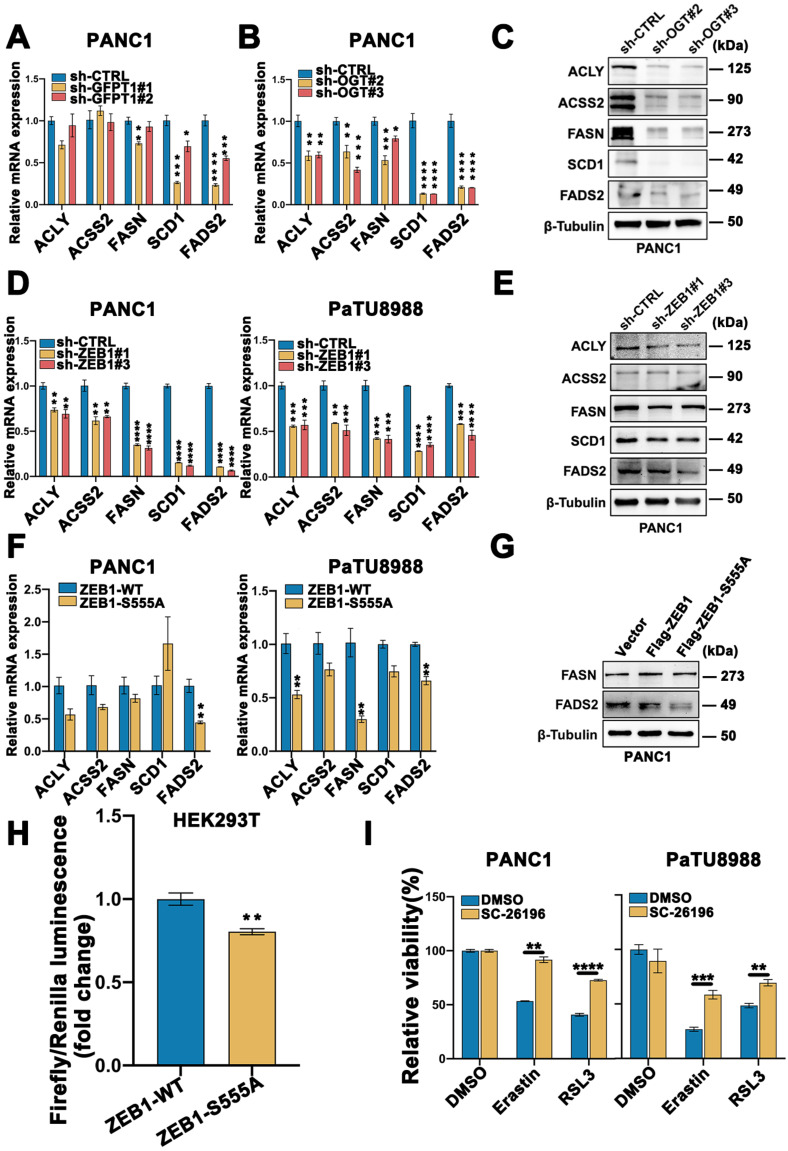
** FASN-FADS2 axis regulated polyunsaturated fatty acid biosynthesis was involved in ZEB1 O-GlcNAcylation driven ferroptosis sensitivity.** (**A-B**) mRNA expression level of ACLY, ACSS2, FASN, SCD1 and FADS2 in sh-GFPT1 PANC1 (**A**) and sh-OGT PANC1 (**B**) cells by RT-qPCR. ACTB was used as a loading control. (**C**) Western blot assessed the expression level of ACLY, ACSS2, FASN, SCD1 and FADS2 in sh-CTRL or sh-OGT PANC1 cells. β-Tubulin was used as a loading control. (**D**) mRNA expression level of ACLY, ACSS2, FASN, SCD1 and FADS2 in ZEB1-silenced (sh-ZEB1) or control (sh-CTRL) PANC1 and PaTU8988 cells. ACTB mRNA expression was used as a loading control. (**E**) Western blot assessed the expression level of ACLY, ACSS2, FASN, SCD1 and FADS2 in sh-CTRL or sh-ZEB1 PANC1 cells. β-Tubulin was used as a loading control. (**F**) RT-qPCR analyzed the expression level of ACLY, ACSS2, FASN, SCD1 and FADS2 in ZEB1-WT and ZEB1-S555A PANC1 and PaTU8988 cells. ACTB mRNA expression was detected as a loading control. (**G**) Western blot analyzed the FASN and FASD2 in ZEB1-WT and ZEB1-S555A PANC1 cells. (**H**) Dual luciferase reporter assay was used to analyze the transcriptional activity of ZEB1-WT and ZEB1-S555A with FADS2 promotor in HEK293T cells. (**I**) CCK-8 assay assessed the cell viability of PANC1 and PaTU8988 cells treated DMSO, Erastin (4, 15 µM) and RSL3 (0.5, 5 µM) with or without SC-26196 (10 µM). RSL3 represents for (1S,3R)-RSL3. Experiments were repeated three times and the data are expressed as the mean ± SEM. ∗* P* < 0.05. ∗∗ *P* < 0.01. ∗∗∗* P* < 0.001. ∗∗∗∗ *P* < 0.0001.

As a critical transcription factor, ZEB1 exerts its biology through regulating transcriptional activity. To further explore the underlying mechanism of ZEB1 O-GlcNAcylation induced mesenchymal pancreatic cancer cell ferroptosis sensitivity, we firstly assessed the lipid metabolism associated enzymes expression level. Western blot analysis showed that the lipid metabolism related enzymes were fully up-regulated in the high glucose condition (**Figure S4C**). To further elucidate the functional role of lipid metabolism in ZEB1 O-GlcNAcylation induced ferroptosis sensitivity, GFPT1 and OGT knockdown cells were used. Compared to the parental cancer cells, FASN, SCD1 and FADS2 expression at the mRNA and protein level consistently downregulated in the GFPT1 and OGT knockdown cells (**Figure 6A-C and Figure S4D**). The similar results can be acquired in ZEB1 knockdown cells (**Figure 6D-E and Figure S4E**).

Concisely, the mRNA and protein expression level of FASN and FADS2 was upregulated in ZEB1 overexpression cancer cells, while downregulated in the ZEB1-S555A mutant cells (**Figure 6F-G and Figure S4F**). FADS2 luciferase activity was inhibited in the ZEB1-S555A mutant cells (**Figure 6H**). Furthermore, to confirm FASN-FADS2 axis isa truly involved in ZEB1 O-GlcNAcylation regulated ferroptosis sensitivity, SC-26196, a classical FADS2 inhibitor, was used. CCK-8 revealed that SC-26196 reversed Erastin and RSL3-induced PANC1 and PaTU8988 cells ferroptosis (**Figure 6I**). These results indicated that ZEB1 O-GlcNAcylation promoted ferroptosis sensitivity through FASN-FADS2 axis.

### ZEB1 O-GlcNAcylation-FASN-FADS2 axis potentiated glucose mediated ferroptosis *in vivo*

**Figure 7:**
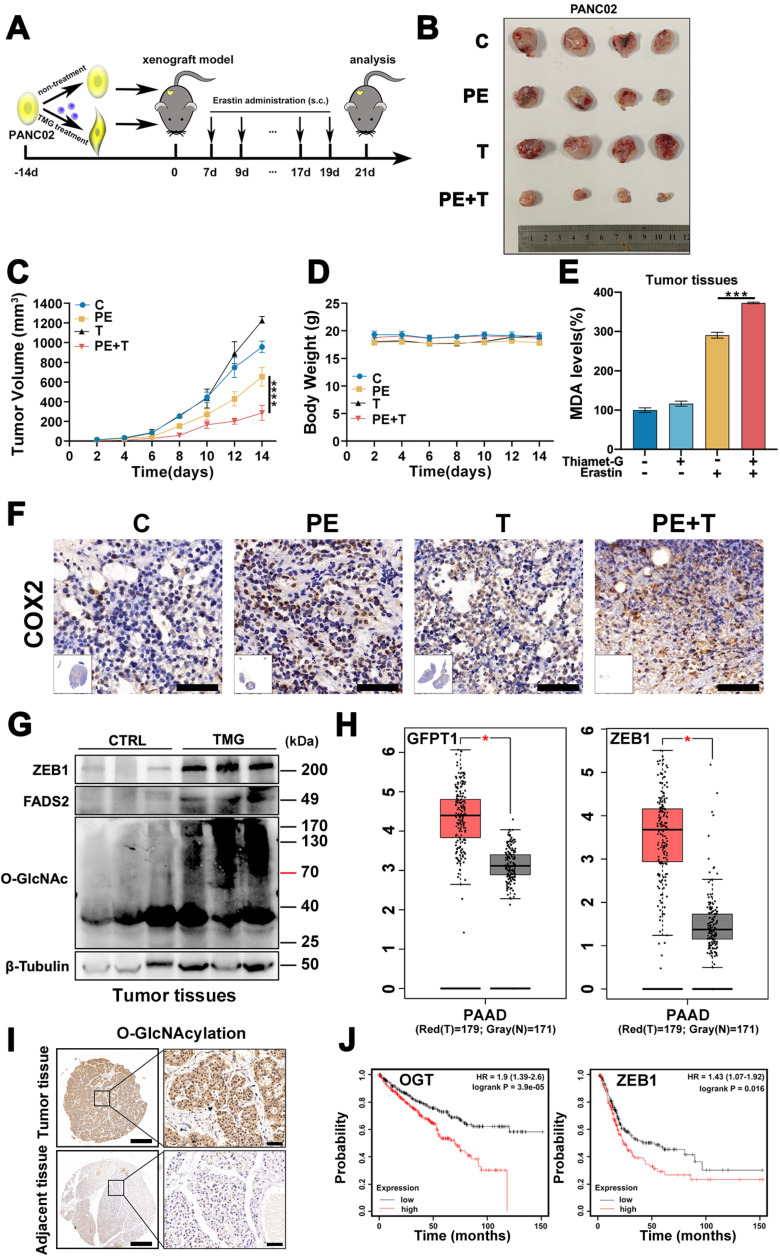
** ZEB1 O-GlcNAcylation-FASN-FADS2 axis potentiated high glucose mediated ferroptosis *in vivo*.** (**A**) Schematic illustration of the therapeutic scheme *in vivo.* PANC02 cells were pre-treated with or without Thiamet-G (20 µM) for 2 weeks and then injected subcutaneously into C57BL/6J mice. The mice were treated with piperazine Erastin (40 mg/kg/s.c.) by injection peritumoral every other day for 2 weeks. (**B**) The tumor image of each group taken out from the sacrificed mice at the end of the study. (**C**) Tumor volumes in mice treated with indicated treatment. (**D**) The body weight of mice in diverse groups as illustrated. (**E**) MDA level in the indicated tumor tissues. (**F**) Representative images of COX2-IHC staining in indicated treatment mice tumors. Scale bar, 50 µm. (**G**) Western blot assessed ZEB1, FADS2 and O-GlcNAcylation expression level in the indicated tumor tissues. (**H**) ZEB1 expression at mRNA levels in 179 PAAD tumor samples and 171 normal controls from the Cancer Genome Atlas (TCGA) database. The data were analyzed by GEPIA. (**I**) Representative images of O-GlcNAcylation-IHC staining in tumors and adjacent tissues from PDAC patients. Scale bar, 500 µm (left) or 50 µm (right). (**J**) Kaplan-Meier curve represented the overall survival of cancer patients. Curve was stratified based on ZEB1 (high 164 samples and low 240 samples) and OGT (high 267 samples and low 263 samples) levels in TCGA database. The data were analyzed by Kaplan-Meier Plotter. C represents for CTRL, PE represents for Piperazine Erastin, T represents for Thiamet-G, PE + T represents for Piperazine Erastin + Thiamet-G, O-GlcNAc represents for O-GlcNAcylation. Experiments were repeated three times and the data are expressed as the mean ± SEM. ∗ *P* < 0.05. ∗∗ *P* < 0.01. ∗∗∗ *P* < 0.001. ∗∗∗∗ *P* < 0.0001.

To validate our *in vitro* observation that ZEB1 O-GlcNAcylation promoted cancer cell ferroptosis sensitivity, we firstly cultured PANC02 cells with or without Thiamet-G and then injected subcutaneously into C57BL/6J mice (**Figure 7A**). When the tumors reached a volume of 50 mm^3^, the mice were treated with Piperazine Erastin (PE), a metabolically stable analog of Erastin for experiment *in vivo*, by peritumoral injection every 2 days for 2 weeks, and tumors were harvested 3 days after the last injection for analysis of ferroptosis.

Compared with vehicle-treated group, TMG pretreatment group exhibited a very mild effect on inhibiting tumor growth and size of PANC02 subcutaneous tumors in C57BL/6 mice, while Piperazine Erastin treatment significantly inhibited the tumor growth and reduced the size by 31.68 % (**Figure 7B**). Indeed, the combination of Erastin and TMG therapy further enhanced the inhibition effect and reduced the size by 69.99 % (**Figure 7C**), without affecting body weight (**Figure 7D**). Moreover, compared to the control group and piperazine Erastin treated group, the combination therapy significantly augmented locally MDA level (**Figure 7E**). IHC revealed the COX2 4, a ferroptosis indicator, expression level significantly increased in the combination therapy group (**Figure 7F**). Also, compared to the control group, pretreatment TMG significantly enhanced ZEB1, O-GlcNAcylation, and FADS2 protein expression levels in the subcutaneous pancreatic carcinoma tissues (**Figure 7G**). Taken together, these results indicated that ZEB1 O-GlcNAcylation regulated lipid metabolism contributed to ferroptotic treatment strategy in pancreatic cancer* in vivo*.

To connect our discovery to clinical application, we evaluated the expression level of ZEB1 and O-GlcNAcylation based on the information in The Cancer Genome Atlas (TCGA) database and found that the expression level of ZEB1 and O-GlcNAcylation was significantly up-regulated in the tumor tissues compared with their adjacent normal tissues in pancreatic tumor (**Figure 7H**). Moreover, immunohistochemical (IHC) staining analyzed the PDAC tissue microarrays revealed that O-GlcNAcylation was predominantly detected in pancreatic tumor, compared with their adjacent tissues (**Figure 7I**). Kaplan-Meier analysis of the TCGA data, with patients divided into high- or low-ZEB1 and O-GlcNAcylation groups, showed a trend of higher overall survival with low ZEB1 and O-GlcNAcylation expression (**Figure 7J**).

## Discussion

**Figure 8:**
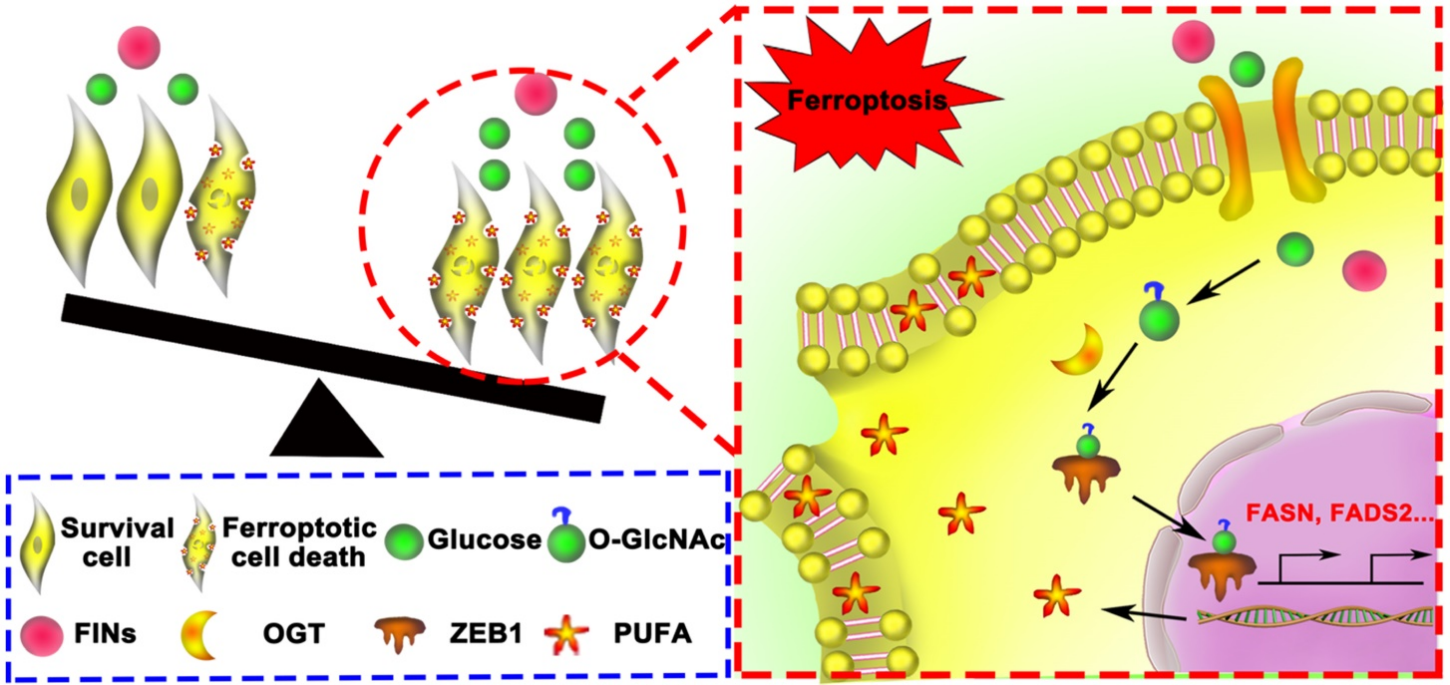
Schematic of glucose triggered ZEB1 O-GlcNAcylation determines mesenchymal pancreatic cancer cell ferroptosis sensitivity.

In this study, we confirmed that glucose could rescue Erastin and RSL3 induced mesenchymal pancreatic cancer cells ferroptosis depending on O-GlcNAcylation modification of ZEB1 *in vitro* and *in vivo* (**Figure 8**). Interestingly, we found glucose activated O-GlcNAc modification of ZEB1 at S555 site, which consequently promoted ZEB1 stabilization and nuclear translocation. Furthermore, ZEB1 O-GlcNAcylation as a promotor of ferroptosis by enhanced the transcriptional ability of the polyunsaturated fatty acid biosynthesis pathway, FASN and FADS2. Therefore, glycolipid metabolism and its related post-translational modification represent for a new therapeutic potentiality of mesenchymal cancer cell ferroptosis and also can be used as a biomarker for predicting the efficacy of ferroptosis in refractory tumor.

Cancer cells acquiring the mesenchymal property, also termed epithelial-mesenchymal transition (EMT), display distinctive cellular characteristics, including stemness, traditional therapy-resistance and the ability to form metastases at distant organs, and thereby contribute to more aggressive behavior of cancer 18, 19. N-cadherin, vimentin, α-smooth muscle actin (α-SMA) and fibroblast activation protein (FAP) are thought to be the gene signatures of mesenchymal type cancer cell, which was also used in this study to identify the mesenchymal cancer cells from a panel of pancreatic cancer cell lines 20. Mesenchymal phenotype of cancer cell is tightly regulated by the key transcription factors, such as ZEB1, Snail, and Slug. In addition to these transcription factors, emerging evidence suggested that ROS was also responsible for acquiring the mesenchymal trait of cancer cell 21. Moreover, therapy resistant mesenchymal cancer cell was closely associated with the disabled antioxidant program. Vasanthi S. Viswanathan *et al*. reported that therapy-resistant high-mesenchymal cell state was more vulnerable to toxic lipid peroxides induced cell death, ferroptosis 3. Our results also confirmed this phenomenon. Therefore, combining redox modulators with conventional therapy may benefit therapeutic efficacy of mesenchymal type cancer cells.

Not all mesenchymal related transcription factors are performance is equally in individual cell types and tissues 22*.* In pancreatic carcinoma, only ZEB1 is highly relevant for tumor onset and progression, especially in regulating cancer cell plasticity and metastasis 23. Moreover, ZEB1 has been shown to play an essential role in cellular lipid metabolism depending on regulating the uptake, accumulation and mobilization of lipids. Recently, ZEB1 has been revealed to provide a bridge between mesenchymal gene expression and lipid peroxide vulnerability. High expression of ZEB1 correlates with the mesenchymal cancer cells sensitivity to ferroptosis in non-small-cell lung cancer cells, head and neck cancer, pancreatic cancer cells and melanoma cells. Our results confirmed that ZEB1 also involved in glucose induced mesenchymal pancreatic cancer ferroptosis sensitivity depending on regulating the polyunsaturated fatty acid biosynthesis pathway. Thus, our study supplies the evidence for ZEB1 as a bridge between the glycolipid metabolism and mesenchymal cancer cell ferroptosis sensitivity. Also, ZEB1 exerts its biology behaviors depending on several layers of epigenetic modification, such as histone modifications, polyadenylation, and methylation 24. Our study firstly found the existed of O-GlcNAc modification of ZEB1 under the high glucose condition.

More and more evidence revealed that increased O-GlcNAcylation levels are associated with pancreatic carcinoma development, metastasis and relapse, ECM remodeling and immune therapy resistance 25, 26. As a non-canonical glycosylation, O-GlcNAcylation is the product of nutrient flux through the hexosamine biosynthetic pathway (HBP), which integrates glucose, amino acid, fatty acid, and nucleotide metabolism 27. Thus, O-GlcNAcylation has been proposed to function as a nutrient sensor regulating cellular processes. In this study, we found O-GlcNAcylation level was also significantly up-regulated accompany with the increased glucose concentration. O-GlcNAcylation involved in a serious of cellar process, including transcription and translation regulation, signal transduction, and metabolism 28. Sp1 O-GlcNAcylation has been shown to modulate its nuclear localization, transactivation and stability 29. O-GlcNAcylation of RelA is facilitated for NF-kB nuclear translocation and transcriptional activity in a context-dependent manner 30. Our study also confirmed O-GlcNAc modification of ZEB1 facilitated its stabilization and nuclear translocation, which is according to the previously publication of O-GlcNAcylation in transcriptional regulation. However, O-GlcNAc modification ZEB1 is a glucose dependent manner or a universal phenomenon still needs further investigation. Moreover, the gene and protein expression mismatch in GFPT1 knockdown cells and OGT knockdown cells, which may be ascribed to GFPT1 is the upstream key enzyme of OGT in hexosamine biosynthesis pathway (HBP) and also involve in glutamine metabolism regulation, a critical metabolic pathway in ferroptosis 31. SLC7A11 chose as the *in vitro* ferroptosis indicator was ascribed to: firstly, SLC7A11 was the core regulator hub that connects glucose metabolism, redox biology, and ferroptosis. Secondly, SLC7A11 expression level was recently reported to be involved in glucose regulated ferroptosis 32. Moreover, GPX4 expression level was unchanged in a glucose concentration dependent manner. The deeper mechanism deserves further investigation.

Taken together, our data demonstrate that disturbed the glycolipid metabolism reprogramming could serve as a good strategy for inducing mesenchymal cancer cell ferroptosis under glucose contexts. Our work also suggests a mechanism for O-GlcNAc modification ZEB1 involving the regulation of glycolipid metabolism as crucial process in mesenchymal pancreatic cancer cell ferroptosis. We suggest that the level of O-GlcNAcylation and ZEB1 may be used as the biomarker to predict the responsiveness of mesenchymal cancer cell to ferroptosis-inducing therapies.

## Conclusions

These results establish a mechanistic link between glycolipid metabolism and O-GlcNAcylation that involved in mesenchymal pancreatic cancer cells ferroptosis susceptibility, which broaden the molecular mechanism of ferroptosis and suggested a potential therapeutic strategy for refractory tumors.

## Supplementary Material

Supplementary figures and tables.

## Abbreviations

ZEB1: zinc finger E-box-binding homeobox 1
CCK-8: Counting Kit-8 kit
shRNA: short hairpin RNA
GEPIA: Gene Expression Profiling Interactive Analysis
FASN: Fatty Synthase
FADS2: Fatty Acid Desaturase 2
TGF: Transforming Growth Factor
RSL3: (1S,3R)-RSL3
DON: 6-Diazo-5-oxo-L-nor-Leucine
UDP-GlcNAc: UDP-GlcNAc Disodium Salt
CHX: Cycloheximide
BADGP: Benzyl 2-acetamido-2-deoxy-a-D-galactopyranoside
TMG: Thiamet G
GFPT1: Glutamine Fructose 6 Phosphate Transaminase 1
OGT: O-Linked N-Acetylglucosamine Transferase
IP: immunoprecipitated
MDA: Malondialdehyde
OGA: O-GlcNAcase
SLC7A11: Solute Carrier Family 7 Member 11
GPX4: Glutathione Peroxidase 4
EMT: Epithelial-Mesenchymal Transition
COX2/PTGS2: Prostaglandin-Endoperoxide Synthase 2
PDAC: Pancreatic Ductal Adenocarcinomas
