# Supplementary material for: O-GlcNAcylation of ZEB1 facilitated mesenchymal pancreatic cancer cell ferroptosis
Source: Int J Biol Sci. 2022 Jun 21;18(10):4135–50. doi: 10.7150/ijbs.71520 (PMC9274488; doi:10.7150/ijbs.71520)
Supplement: Supplementary file 1 — Supplementary figures and tables. [file ijbsv18p4135s1.pdf]

Supplementary Material

Glucose triggered ZEB1 O-GlcNAcylation determines mesenchymal pancreatic cancer cell ferroptosis sensitivity

Xin Wang[1,2#], Mengqi Liu[3#], Yue Chu[4#], Yanfang Liu[3], Xiongfeng Cao[2], Han Zhang[1], Yao Huang[3], Aihua Gong[3], Xiang Liao[5*], Dongqing Wang[2*], Haitao Zhu[1,2*]

1. Laboratory of Medical Imaging, Affiliated Hospital of Jiangsu University, Zhenjiang, China, 212001
2. Department of Medical Imaging, Affiliated Hospital of Jiangsu University, Zhenjiang, China, 212001
3. School of Medicine, Jiangsu University, Zhenjiang, China, 212013
4. Department of Medical Imaging, Affiliated Hospital of Nanjing Medicine University, 211166
5. Department of Laboratory Medicine, Affiliated Hospital of Jiangsu University, Zhenjiang, China, 212001

# These authors contributed equally: Xin Wang, Mengqi Liu, Yue Chu.

*Correspondence should be addressed to Xiang Liao; liaoxiang025@126.com, Dongqing Wang; wangdongqing71@163.com, and Haitao Zhu; zhht25@163.com

Figure S1. Glucose enhanced mesenchymal pancreatic cancer cells ferroptosis sensitivity and O-GlcNAcylation level. (**A**) Western blot showed the basic expressions of ZEB1, E-Cadherin and N-Cadherin in PANC1, PaTU8988, BXPC3, ASPC1 and PANC02 cells. β-Tubulin protein expression was detected as a loading control. (**B**) The cell viability of PANC1 and PaTU8988 cells with the treatments DMSO, Erastin (4, 15 μM) or RSL3 (0.5, 5 μM) for 48 h combined with Ferrostain-1 (5 μM), Z-VAD-FMK (1 μM) or Necrosulfonamide (0.5 μM) were monitored using a CCK-8 assay. The relative viability was normalized to DMSO control. (**C**) MDA level in PaTU8988 cells treated with DMSO or Erastin (15 μM) for 48 h in glucose concentration gradient medium (4500, 2750, 1875, 1000 mg/mL). (**D**) Western blot analyzed the ZEB1 and SLC7A11 expression at the protein level in PANC1 and PaTU8988 cells cultured with glucose concentration gradient medium (4500, 3625, 2750, 1875, 1000 mg/mL). β-Tubulin protein expression was detected as a loading control. (**E-F**) Western blot analyzed the O-GlcNAc expression at the protein level in PANC1 (**E**) and PaTU8988 (**F**) cells cultured with glucose concentration gradient medium (4500, 3625, 2750, 1875, 1000 mg/mL). β-Tubulin protein expression was detected as a loading control. (**G**) Western blot showed the O-GlcNAcylation and SLC7A11 level in HPDE6 and PANC1 cells cultured with glucose or glucose-depletion medium for 48 h. β-Tubulin was used as a loading control. (**H**) MDA level in PANC1 and HPDE6 cells treated with DMSO or Erastin (4 μM) for 48 h in glucose present or depletion medium. (**I**) Western blot showed the O-GlcNAcylation level in PANC1, MCF7 and HT1080 cells cultured with glucose present medium for 48 h. β-Tubulin was used as a loading control. (**J**) CCK-8 assay assessed the cell viability of HT1080 and MCF7 cells treated with Erastin concentration gradient for 48h in glucose present medium, respectively. RSL3 represents for (1S,3R)-RSL3. MDA represents for malondialdehyde. O-GlcNAc represents for

O-GlcNAcylation. Experiments were repeated three times and the data are expressed as the mean ± SEM. * $P < 0.05$. ** $P < 0.01$. *** $P < 0.001$. **** $P < 0.0001$.

Figure S2. Glucose induced O-GlcNAcylation promoted mesenchymal pancreatic cancer cells ferroptosis. (**A**) PANC1 and PaTU8988 cells were transfected with sh-CTRL or sh-GFPT1 plasmids and GFPT1 protein expression were measured by immunoblotting analysis. β-Tubulin protein expression was detected as a loading control. (**B**) PANC1 and PaTU8988 cells were transfected with sh-CTRL or sh-OGT plasmids and OGT expression were measured by immunoblotting analysis. β-Tubulin protein expression was detected as a loading control. (**C**) Immunoblotting images of O-GlcNAcylation in sh-CTRL or sh-GFPT1 PANC1 and PaTU8988 cells. β-Tubulin protein expression was detected as a loading control. (**D**) Immunoblotting images of O-GlcNAcylation in sh-CTRL or sh-OGT PANC1 and PaTU8988 cells. β-Tubulin protein expression was detected as a loading control. (**E**) Western Blot assessed the SLC7A11 and GPX4 levels in GFPT1-silenced (sh-GFPT1) or control (sh-CTRL) PaTU8988 cells. β-Tubulin was used as a loading control. (**F**) The panel showed the ratios between SLC7A11/GPX4 and β-Tubulin protein levels of Figure S2E, and the sh-CTRL cells were set to 100%. (**G**) The panel showed the ratios between SLC7A11/GPX4 and β-Tubulin protein levels of Figure 2C, and the sh-CTRL cells were set to 100%. (**H**) Western Blot assessed the SLC7A11 and GPX4 levels in OGT-silenced (sh-OGT) or control (sh-CTRL) PaTU8988 cells. β-Tubulin was used as a loading control. (**I**) Immunoblotting images of O-GlcNAcylation in PANC1 and PaTU8988 cells with or without DON treatment for 48h. β-Tubulin protein expression was detected as a loading control. (**J**) Immunoblotting images of O-GlcNAcylation in PANC1 and PaTU8988 cells with BADGP (5 mM)

or Thiamet-G (20 μM) for 48h. β-Tubulin protein expression was detected as a loading control. O-GlcNAc represents for O-GlcNAcylation. DON represents for 6-Diazo-5-oxo-L-nor-Leucine. BADGP represents for Benzyl 2-acetamido-2-deoxy-a-D-galactopyranoside.

Figure S3. ZEB1 O-GlcNAcylation is involved in glucose regulated mesenchymal pancreatic cancer cells ferroptosis sensitivity. (**A**) The correlation between EMT-TFs and O-GlcNAcylation-associated genes expression was detected from the Cancer Genome Atlas (TCGA) PDAC samples. The data were analyzed by GEPIA. (**B**) PaTU8988 cells were transfected with sh-CTRL or sh-ZEB1 plasmids. Cell lysates were prepared to analyze the expression levels of the ZEB1 by Western blot. β-Tubulin protein expression was detected as a loading control. (**C**) Western blot assessed SLC7A11 and GPX4 levels in ZEB1-silenced (sh-ZEB1) or control (sh-CTRL) PaTU8988 cells. β-Tubulin was used as a loading control. (**D**) MDA level in ZEB1-silenced (sh-ZEB1) or control (sh-CTRL) PaTU8988 cells treated with Erastin (15 μM). (**E**) ZEB1 O-GlcNAcylation was determined by Co-IP in PANC1 cells. (**F**) RT-qPCR showed the mRNA expression of ZEB1 in PANC1 and PaTU8988 cells with or without OGT silenced. ACTB mRNA expression was detected as a loading control. (**G**) PANC1 and PaTU8988 cells were treated with BADGP (5 mM), Thiamet-G (20 μM) or DMSO for 48 h. Cell lysates were prepared to analyze the expression levels of the ZEB1 by Western blot. β-Tubulin protein expression was detected as a loading control. EMT-TFs represents for Epithelial-mesenchymal transition- associated transcription factors. PDAC represents for Pancreatic Ductal Adenocarcinoma. BADGP represents for Benzyl 2-acetamido-2-deoxy-a-D-galactopyranoside. Experiments were repeated three times and the data are expressed as the mean ± SEM.

Figure S4. FASN-FADS2 axis regulated polyunsaturated fatty acid biosynthesis was involved in ZEB1 O-GlcNAcylation driven ferroptosis sensitivity. (**A**) Co-IP analysis of ZEB1 O-GlcNAcylation in PANC1 and PaTU8988 cells transfected with Vector, ZEB1-WT-Flag, ZEB1-T678A-Flag and ZEB1-S555A-Flag plasmids. β-Tubulin protein expression was detected as a loading control. (**B**) PaTU8988 cells were transfected with indicated plasmids, followed by Erastin (15 μM) treatment for 48h. Relative lipid peroxidation levels was measured through MDA assay kit. (**C**) PANC1 and PaTU8988 cells were cultured in normal or glucose-depletion medium. Cell lysates were prepared to analyze the expression levels of ACLY, ACSS2, FASN, SCD1 and FADS2 proteins by Western blot. β-Tubulin protein expression was detected as a loading control. (**D**) Western blot assessed the expression level of ACLY, ACSS2, FASN, SCD1 and FADS2 in sh-CTRL or sh-OGT PaTU8988 cells. β-Tubulin was used as a loading control. (**E**) Western blot assessed the expression level of ACLY, ACSS2, FASN, SCD1 and FADS2 in sh-CTRL or sh-ZEB1 PaTU8988 cells. β-Tubulin was used as a loading control. (**F**) Western blot analyzed the FASN and FASD2 in ZEB1-WT and ZEB1-S555A PaTU8988 cells. β-Tubulin was used as a loading control. O-GlcNAc represents for O-GlcNAcylation. MDA represents for malondialdehyde. Experiments were repeated three times and the data are expressed as the mean ± SEM. * $P < 0.05$. ** $P < 0.01$. *** $P < 0.001$. **** $P < 0.0001$.

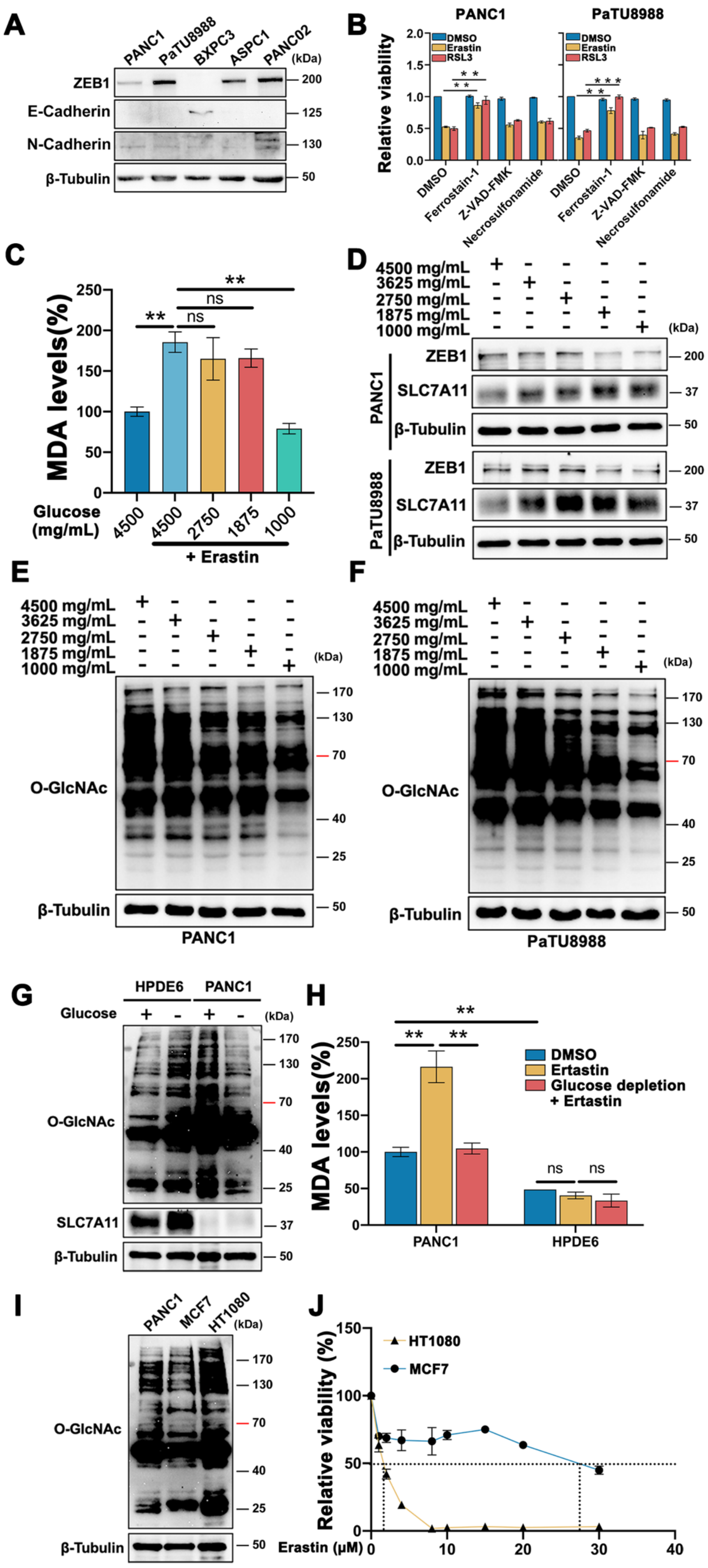
A
PANC1
PaTU8988
BXPC3
ASPC1
PANC02
(kDa)
ZEB1
E-Cadherin
N-Cadherin
β-Tubulin
200
125
130
50
B
PANC1
PaTU8988
DMSO
Erastin
RSL3
Relative viability
DMSO
Ferrostain-1
Z-VAD-FMK
Necrosulfonamide
C
MDA levels(%)
Glucose (mg/mL)
4500
2750
1875
1000
+ Erastin
ns
D
4500 mg/mL
3625 mg/mL
2750 mg/mL
1875 mg/mL
1000 mg/mL
SLC7A11
PANC1
PaTU8988
37
E
O-GlcNAc
170
130
70
40
25
F
G
HPDE6
Glucose
H
DMSO
Ertastin
Glucose depletion
+ Ertastin
HPDE6
I
MCF7
HT1080
J
Relative viability (%)
Erastin (μM)

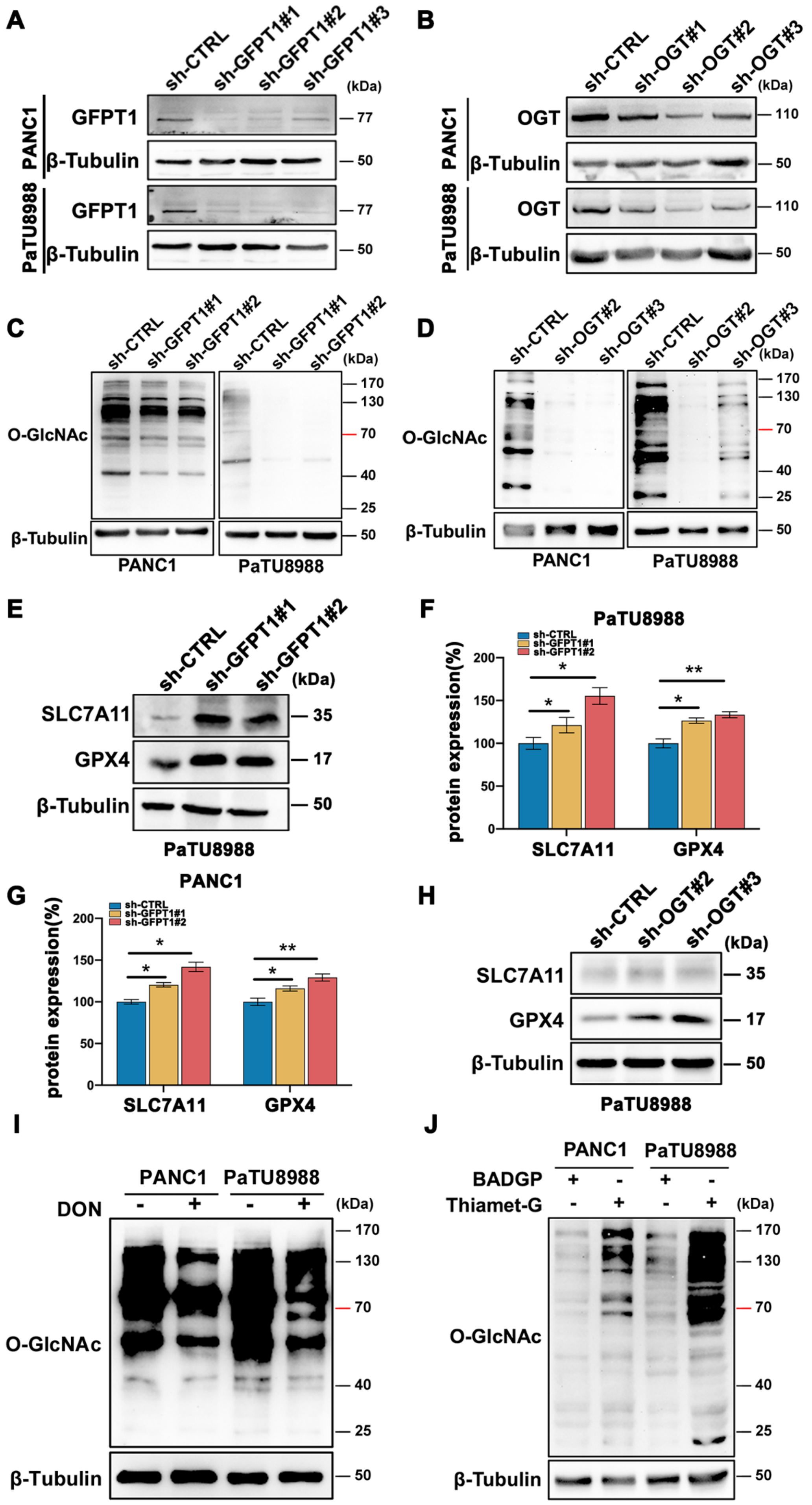
A
sh-CTRL
sh-GFPT1#1
sh-GFPT1#2
sh-GFPT1#3
(kDa)
PANC1
PaTU8988
GFPT1
β-Tubulin
77
50
B
sh-CTRL
sh-OGT#1
sh-OGT#2
sh-OGT#3
OGT
110
C
O-GlcNAc
170
130
70
40
25
D
sh-OGT#2
sh-OGT#3
E
SLC7A11
GPX4
35
17
F
G
protein expression(%)
200
150
100
50
0
*
**
H
I
DON
-
+
J
BADGP
Thiamet-G

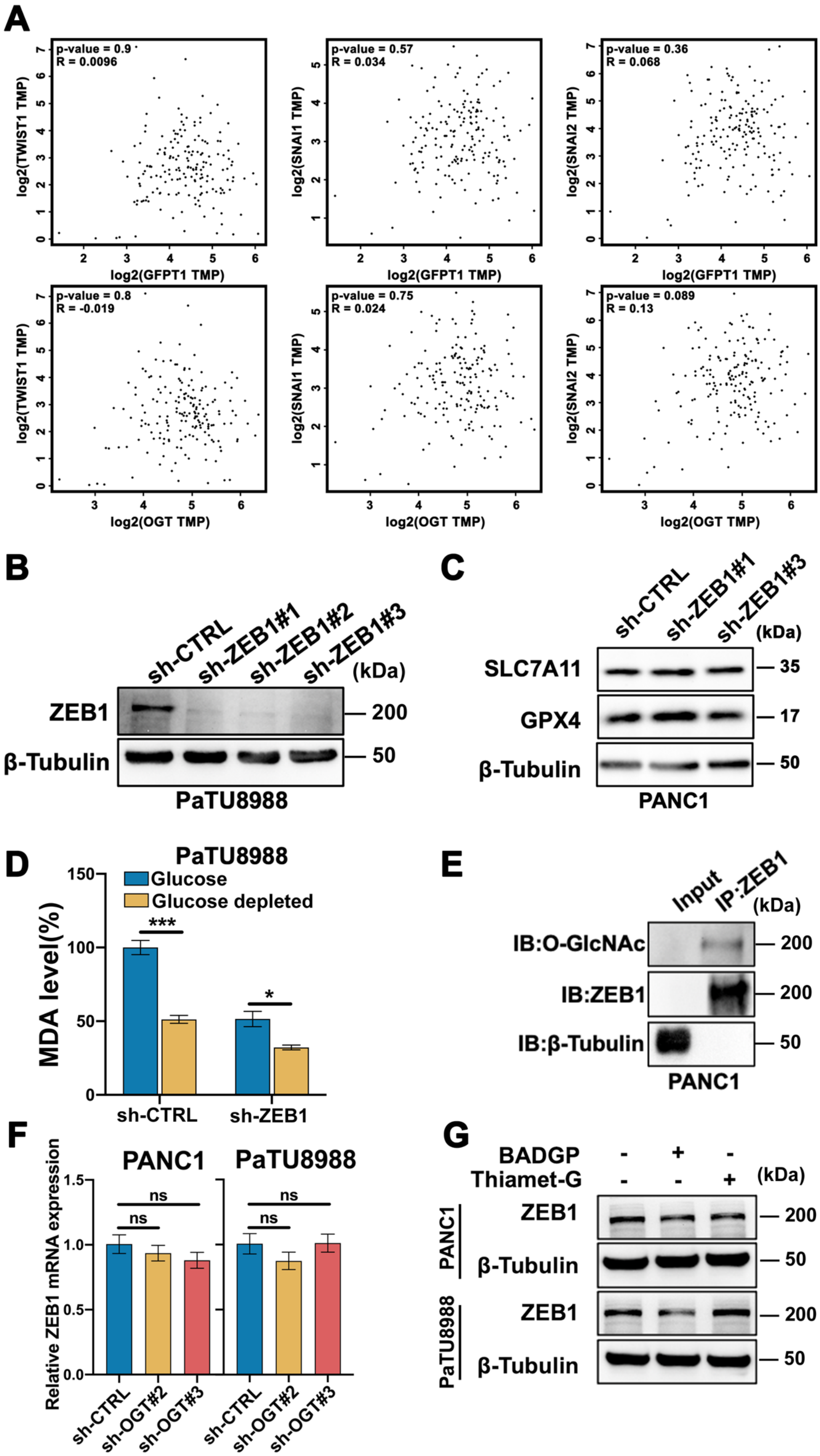
A
p-value = 0.9
R = 0.0096
log2(TWIST1 TMP)
log2(GFPT1 TMP)
p-value = 0.57
R = 0.034
log2(SNAI1 TMP)
log2(GFPT1 TMP)
p-value = 0.36
R = 0.068
log2(SNAI2 TMP)
log2(GFPT1 TMP)
p-value = 0.8
R = -0.019
log2(TWIST1 TMP)
log2(OGT TMP)
p-value = 0.75
R = 0.024
log2(SNAI1 TMP)
log2(OGT TMP)
p-value = 0.089
R = 0.13
log2(SNAI2 TMP)
log2(OGT TMP)
B
sh-CTRL
sh-ZEB1#1
sh-ZEB1#2
sh-ZEB1#3
(kDa)
ZEB1
200
β-Tubulin
50
PaTU8988
C
sh-CTRL
sh-ZEB1#1
sh-ZEB1#3
(kDa)
SLC7A11
35
GPX4
17
β-Tubulin
50
PANC1
D
PaTU8988
Glucose
Glucose depleted
MDA level(%)
***
*
sh-CTRL
sh-ZEB1
E
Input
IP:ZEB1
(kDa)
IB:O-GlcNAc
200
IB:ZEB1
200
IB:β-Tubulin
50
PANC1
F
PANC1
PaTU8988
Relative ZEB1 mRNA expression
ns
ns
ns
ns
sh-CTRL
sh-OGT#2
sh-OGT#3
sh-CTRL
sh-OGT#2
sh-OGT#3
G
BADGP - + -
Thiamet-G - - +
(kDa)
PANC1
ZEB1
200
β-Tubulin
50
PaTU8988
ZEB1
200
β-Tubulin
50

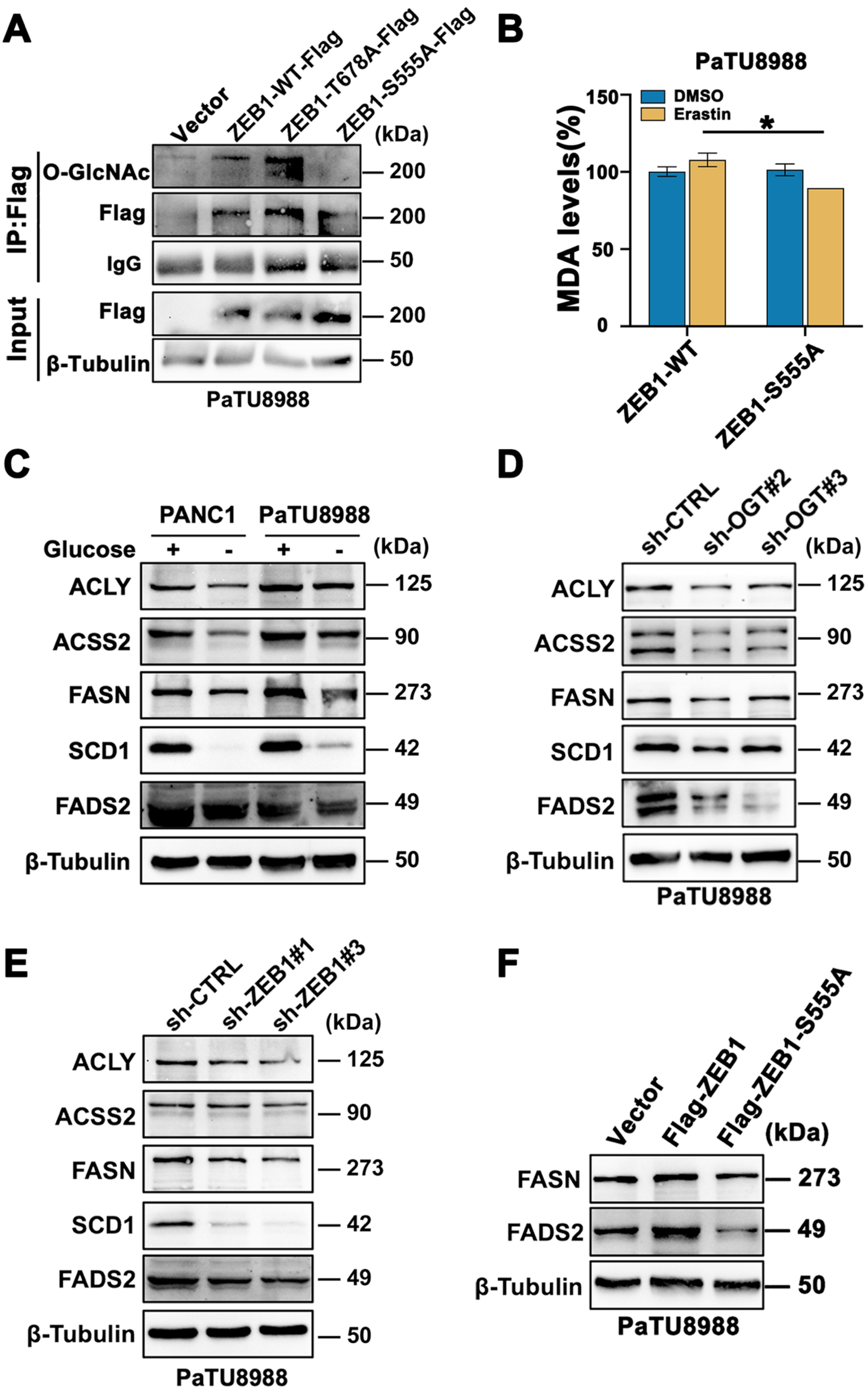
A
Vector
ZEB1-WT-Flag
ZEB1-T678A-Flag
ZEB1-S555A-Flag
(kDa)
IP:Flag
O-GlcNAc
200
Flag
200
IgG
50
Input
Flag
200
β-Tubulin
50
PaTU8988
B
PaTU8988
DMSO
Erastin
MDA levels(%)
150
100
50
0
*
ZEB1-WT
ZEB1-S555A
C
PANC1
PaTU8988
Glucose
+
-
+
-
(kDa)
ACLY
125
ACSS2
90
FASN
273
SCD1
42
FADS2
49
β-Tubulin
50
D
sh-CTRL
sh-OGT#2
sh-OGT#3
(kDa)
ACLY
125
ACSS2
90
FASN
273
SCD1
42
FADS2
49
β-Tubulin
50
PaTU8988
E
sh-CTRL
sh-ZEB1#1
sh-ZEB1#3
(kDa)
ACLY
125
ACSS2
90
FASN
273
SCD1
42
FADS2
49
β-Tubulin
50
PaTU8988
F
Vector
Flag-ZEB1
Flag-ZEB1-S555A
(kDa)
FASN
273
FADS2
49
β-Tubulin
50
PaTU8988

Table S1 Primers for knockdown plasmids

Gene	Sequence
sh-CTRL	5`-GTTCTCCGAACGTGTCACGTT-3`
sh-GFPT1#1	5`-GCAGATACTTTGATGGGTCTT-3`
sh-GFPT1#2	5`-CGTCTTTCTATCCATCGAATT-3`
sh-GFPT1#3	5`-CCTCTGGCTTTGGTGGATAAA-3`
sh-OGT#1	5`-TTTAGCACTCTGGCAATTAAA-3`
sh-OGT#2	5`-GCTGAGCAGTATTCCGAGAAA-3`
sh-OGT#3	5`-GCCCTAAGTTTGAGTCCAAAT-3`
sh-ZEB1#1	5`-GCGGTAGATGGTAATGTAATA-3`
sh-ZEB1#2	5`-GCAAGTGTTGGAGAATAATCA-3`
sh-ZEB1#3	5`-GCATACACCTACTCAACTACG-3`

Table S2 Primers for overexpression and mutant plasmids

Gene	Target sequence
ZEB1-WT-Flag	F:5`-TCCTCGAGACTAGTTgccaccatgaaagttacaaattataatactgtggtag-3`
	R:5`-GTCCATTCCGCGGCCGCTggcttcatttgtcttttcttcagacac-3`
ZEB1-T678A-Flag	F:5`-tcCTCGAGACTAGTTgccaccatgaaagttacaaattataatactgtggtag-3`
	R:5`-GTCCATTCCGCGGCCGCTggcttcatttgtcttttcttcagac-3`
ZEB1-S555A-Flag	F:5`-TCCTCGAGACTAGTTgccaccatgaaagttacaaattataatactgtggtag-3`
	R:5`-GTCCATTCCGCGGCCGCTggcttcatttgtcttttcttcagacac-3`

Table S3 Primers for RT-qPCR

Gene	Sequence
ACTB	F: 5`-CACCATTGGCAATGAGCGGTTC-3`
	R: 5`-AGGTCTTTGCGGATGTCCACGT-3`
ZEB1	F:5`-GGCATACACCTACTCAACTACGG-3`
	R:5`-TGGGCGGTGTAGAATCAGAGTC-3`
ACLY	F: 5`-ATCGGTTCAAGTATGCTCGGG-3`
	R: 5`-GACCAAGTTTTCCACGACGTT-3`
ACSS2	F: 5`-AAAGGAGCAACTACCAACATCTG-3`
	R: 5`-GCTGAACTGACACACTTGGAC-3`
FASN	F:5`-TTCTACGGCTCCACGCTCTTCC-3`
	R:5`-GAAGAGTCTTCGTCAGCCAGGA-3`
SCD1	F: 5`-CCTGGTTTCACTTGGAGCTGTG-3`
	R: 5`-TGTGGTGAAGTTGATGTGCCAGC-3`
FADS2	F:5`-TGCAACGTGGAGCAGTCCTTCT-3`
	R:5`-GGCACATAGAGACTTCACCAGC-3`

Table S4 Antibodies for Western blot, IP and IHC

Antibody	Source	Catalog number	Application (dilution)
O-Linked N-Acetylglucosamine	Abcam	ab201995	WB (1:1000)<br>IHC (1:200)

β-Tubulin	ABclonal	AC030	WB (1:5000)
ZEB1	CST	3396S	WB (1:1000)
ZEB1	Proteintech	21554-1-AP	IP (1:200), IHC (1:50)
E-Cadherin	Proteintech	20874-1-AP	WB (1:1000)
N-Cadherin	Proteintech	22018-1-AP	WB (1:1000)
SLC7A11	CST	12691S	WB (1:1000)
GFPT1	Proteintech	14132-1-AP	WB (1:1000)
GPX4	Abcam	ab125066	WB (1:1000)
OGT	Proteintech	11576-2-AP	WB (1:1000)
HDAC	GeneTex	GTX100513	WB (1:1000)
FASN	CST	3189S	WB (1:1000)
ACLY	CST	4332S	WB (1:1000)
ACSS2	Santa cruz	SC-398559	WB (1:1000)
FADS2	Proteintech	28034-1-AP	WB (1:1000)
SCD1	Abcam	ab236868	WB (1:1000)
COX2	Abcam	ab283574	IHC (1:50)
Flag	ABclonal	AE005	IP (1:200)

Abbreviations: WB, western blotting assay; CST, Cell Signaling Technology.
